# Single cell RNA sequencing reveals human tooth type identity and guides *in vitro* hiPSC derived odontoblast differentiation (iOB)

**DOI:** 10.3389/fdmed.2023.1209503

**Published:** 2023-07-20

**Authors:** Sesha Hanson-Drury, Anjali P. Patni, Deborah L. Lee, Ammar Alghadeer, Yan Ting Zhao, Devon Duron Ehnes, Vivian N. Vo, Sydney Y. Kim, Druthi Jithendra, Ashish Phal, Natasha I. Edman, Thomas Schlichthaerle, David Baker, Jessica E. Young, Julie Mathieu, Hannele Ruohola-Baker

**Affiliations:** ^1^ Department of Oral Health Sciences, School of Dentistry, University of Washington, Seattle, WA, United States; ^2^ Department of Biochemistry, School of Medicine, University of Washington, Seattle, WA, United States; ^3^ Institute for Stem Cell and Regenerative Medicine, School of Medicine, University of Washington, Seattle, WA, United States; ^4^ Department of Genetic Engineering, SRM Institute of Science and Technology, Chennai, India; ^5^ Department of Biomedical Dental Sciences, College of Dentistry, Imam Abdulrahman bin Faisal University, Dammam, Saudi Arabia; ^6^ Department of Biology, University of Washington, Seattle, WA, United States; ^7^ Department of Biotechnology, SRM Institute of Science and Technology, Chennai, India; ^8^ Department of Bioengineering, University of Washington, Seattle, WA, United States; ^9^ Institute for Protein Design, University of Washington, Seattle, WA, United States; ^10^ Molecular and Cellular Biology Graduate Program, University of Washington, Seattle, WA, United States; ^11^ Medical Scientist Training Program, University of Washington, Seattle, WA, United States; ^12^ Department of Laboratory Medicine and Pathology, University of Washington, Seattle, WA, United States; ^13^ Department of Comparative Medicine, School of Medicine, University of Washington, Seattle, WA, United States

**Keywords:** odontoblast, single cell RNA sequencing, enamel knot, cell signaling, de novo designed mini protein binders, human tooth, regenerative dentistry, tooth development

## Abstract

Over 90% of the U.S. adult population suffers from tooth structure loss due to caries. Most of the mineralized tooth structure is composed of dentin, a material produced and mineralized by ectomesenchyme derived cells known as odontoblasts. Clinicians, scientists, and the general public share the desire to regenerate this missing tooth structure. To bioengineer missing dentin, increased understanding of human tooth development is required. Here we interrogate at the single cell level the signaling interactions that guide human odontoblast and ameloblast development and which determine incisor or molar tooth germ type identity. During human odontoblast development, computational analysis predicts that early FGF and BMP activation followed by later HH signaling is crucial. Here we generate a differentiation protocol based on this sci-RNA-seq analysis to produce mature hiPSC derived odontoblasts in vitro (iOB). Further, we elucidate the critical role of FGF signaling in odontoblast maturation and its biomineralization capacity using the *de novo* designed FGFR1/2c isoform specific minibinder scaffolded as a C6 oligomer that acts as a pathway agonist. Using computational tools, we show on a molecular level how human molar development is delayed compared to incisors. We reveal that enamel knot development is guided by FGF and WNT in incisors and BMP and ROBO in the molars, and that incisor and molar ameloblast development is guided by FGF, EGF and BMP signaling, with tooth type specific intensity of signaling interactions. Dental ectomesenchyme derived cells are the primary source of signaling ligands responsible for both enamel knot and ameloblast development.

## 1. Introduction

Untreated dental caries is the most prevalent disease globally, with the Center for Disease Control finding that 90% of adults in the United States (U.S.) suffer from dental caries (1). Further, dental pulp disease was the primary diagnosis for over 400,000 emergency department visits in the U.S. (2), highlighting the need for significant resources to restore both the dental pulp and the mineralized dentin tooth structure it produces. The current method to return form and function to the lost tooth structure with artificial prosthesis such as fillings and crowns can initiate a continuous cycle of restoration replacement, each replacement leading to increased tooth structure loss due to preparation requirements, recurrent caries, or fracture (3). This process, known clinically as the “tooth cycle of death,” can ultimately lead to tooth removal and replacement with a dental implant, currently one of the best tooth alternatives. Importantly, after 9 years, 45% of dental implants develop peri-implantitis (4), an inflammatory process that can lead to loss of the implant and surrounding bone. At this stage the patient often suffers from insufficient bone levels to support a new dental implant, leaving both the patient and clinician in a treatment quandary. Regenerative dentistry seeks to produce stem cell tools to regenerate missing tooth structure. The need for a tooth organoid is paramount.

Reciprocal and continual signaling interactions between the cells of the dental ectomesenchyme and dental epithelium are required for tooth formation (5), disruption of which arrests tooth development (6). Multiple signaling pathways are active throughout tooth development including fibroblast growth factor (FGF), bone morphogenic protein (BMP), hedgehog (HH), and wingless/integrated (WNT) (7, 8). Recently, single cell analysis of the developing human oral cavity revealed that transforming growth factor beta (TGFβ), neurotrophin (NT), HH, BMP, and WNT play critical roles in ameloblast development (9). However, the specific cells of the dental pulp involved in these signaling interactions and their impact on ameloblast development remains unknown.

The majority of mineralized tooth structure is composed of dentin, a vital tissue produced and mineralized by odontoblasts (OB), overlaid by a coat of enamel synthesized by ameloblasts. Murine OB development has been characterized in detail (10). However, mice constantly replenish missing tooth structure through several stem cell niches absent in human teeth, posing translational challenges between the species. Human OB differentiation and maturation remains largely unknown due to the rarity of fetal tissue samples. Importantly, the recent single cell sequencing of the developing human OB lineage (9) now licenses a deeper understanding of OB differentiation towards regenerative dentistry.

Beyond guiding cellular lineage commitment and differentiation, intercellular signaling also shapes the type of tooth that is formed (e.g., incisor or molar). At the early bud stage, odontogenic potential shifts from the overlying dental epithelium to the neural crest derived dental ectomesenchyme (11). In mice, it has been shown that determination of tooth identity is regulated by the dental ectomesenchyme derived cells (6). The dental epithelium derived enamel knot acts as a signaling center that triggers cell proliferation and cytodifferentiation of the dental papilla during tooth morphogenesis (12). Importantly, our lab recently identified FGF4 as a biomarker for the human enamel knot (9). Yet in humans the dental cell types and the intercellular signaling patterns that shape crown morphology, and therefore tooth type, remains unknown.

We have previously generated a single cell combinatorial indexing RNA sequencing (sci-RNA-seq) atlas of the developing human tooth germ, identifying the dental ectomesenchyme and dental epithelium derived cell types comprehensively (9). Here we interrogate at the single cell level the signaling interactions that guide human odontoblast and ameloblast development and that determine incisor or molar tooth germ type identity. Further, we apply the sci-RNA-seq predicted signaling pathways to generate a hiPSC derived odontoblast differentiation method (iOB) using *de novo* designed FGFR1/2 c-isoform mini protein binders to produce a tool for regenerative dentistry therapeutics and disease modeling goals.

## 2. Materials and methods

### 2.1. Single cell RNA sequencing of human fetal tooth germs

#### 2.1.1. Single cell combinatorial indexing RNA sequencing

As described previously (9), 312 human fetal toothgerm samples (201 incisors, 111 molars) and 22 fetal jaw segments (10 anterior, 12 posterior) were collected from 5 gestational week (gw) groups representing the following developmental stages for deciduous tooth differentiation: the bud stage (9–11gw), cap stage (12–13gw), early bell stage (14–16gw), and late bell stage (17–22gw) (7, 13). Due to the limited number of samples obtained for 14–16gw, molar and incisor tissues were combined prior to single cell RNA sequencing. sci-RNA-seq was performed in collaboration with the Brotman Baty Institute (14). Low quality reads were removed by filtering UMI reads higher than 1,500 and lower than 100, followed by removal of all mitochondrial reads. Utilizing the Monocle 3 workflow (15, 16) data underwent normalization by size factor, preprocessing, dimension reduction into Uniform Manifold Approximation and Projection (UMAP) space (17), and unsupervised clustering producing grouping of the cells into clusters based on similarity of gene expression (14). Cell cycle effect was regressed out (18) by simple linear regression using Seurat (19). Cell lineage trajectories and pseudotime were produced using Monocle 3. Pseudotime is calculated from dynamic changes in differentially expressed genes (DEG) and defines a cell's progress along a developmental trajectory (15).

#### 2.1.2. Identification of critical signaling pathways via the Top Pathways analysis

**Figure 1:**
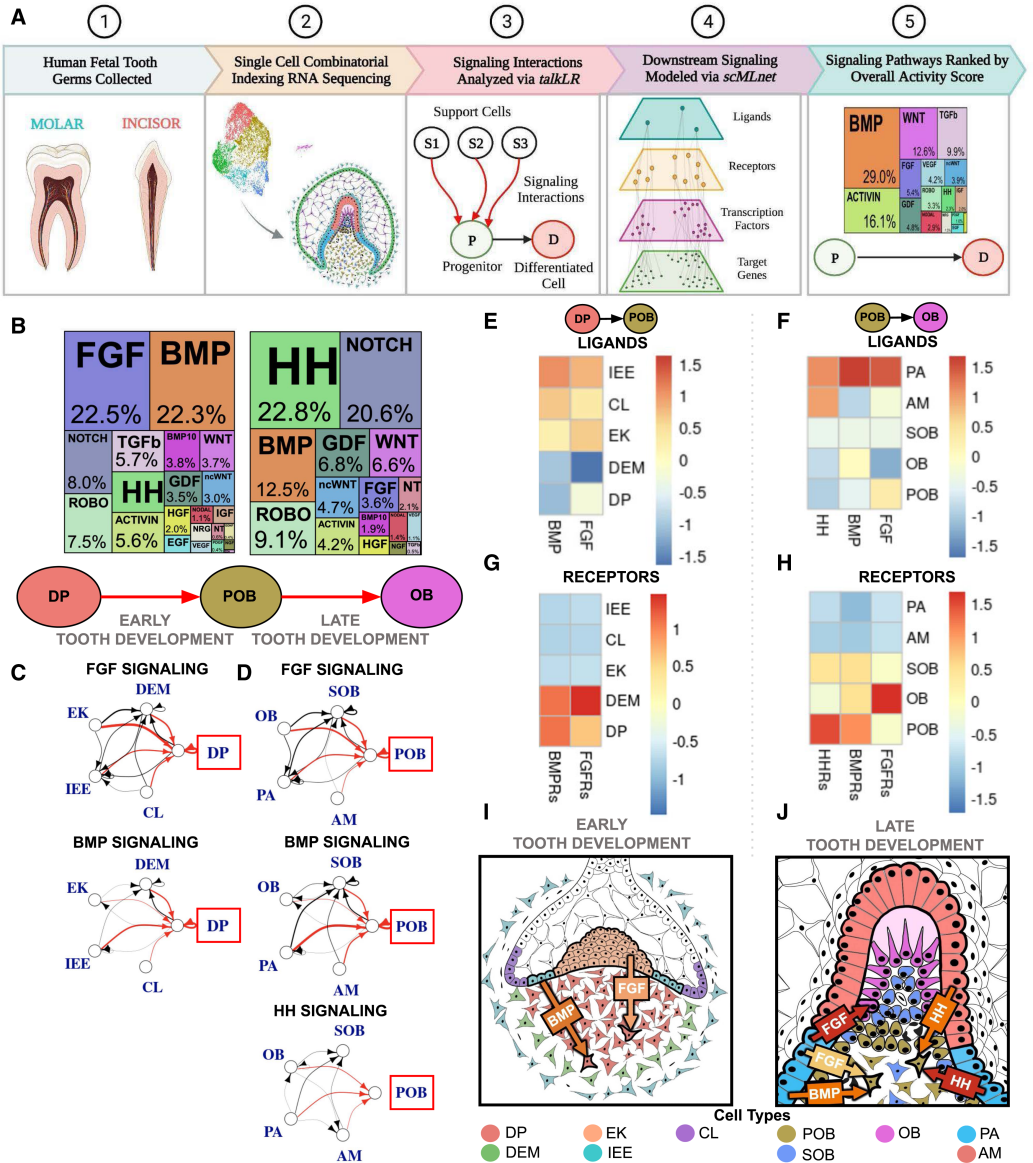
Single cell RNA sequencing of fetal tooth germ predicts FGF, BMP, and HH signaling are critical to human odontoblast development. (**A**) Experimental and computational workflow for single cell combinatorial indexing RNA sequencing and Top Pathways analysis of developing human toothgerms. (**B**) Downstream signaling pathways ranked by activity in odontoblast development indicate FGF and BMP are critical to the dental papilla (DP) as it transitions to preodontoblast (POB); HH, BMP and NOTCH are the most active as POB transitions to odontoblast (OB). The sources of critical signaling ligands for the Top Pathways involved for DP to POB (**C**) and POB to OB (**D**) originate from both the dental epithelium and mesenchyme derived tissues. The number of ligand-receptor interactions denoted by the thickness of the line, arrowheads indicating the cell possessing the receptor, and interactions of interest (red) and between support cells (black), with the progenitor of interest in the red box. Heatmaps were generated by aggregating pathway ligand and receptor gene expression for the DP to POB (**E,G**) and POB to OB (**F,H**) transitions, averaged per cluster. (**I**) Summary schematic depicting early human tooth development, where it is predicted that the majority of FGF and BMP signaling ligands are produced by the dental epithelium derived enamel knot (EK) and inner enamel epithelium (IEE), respectively, which bind to receptors on the DP. (**J**) At late human tooth development, the dental epithelium derived preameloblast (PA) and ameloblast (AM) produce much of the FGF, BMP and HH signaling ligands and bind to receptors on the POB. The color of arrow denotes expression of signaling ligands and receptors (yellow: mild; orange: moderate; red: high). Note that this image is an inset of the whole tooth germ focused on the incisal edge. non-canonical WNT (ncWNT). Graphics generated using InkScape and Biorender.

Our lab has developed a comprehensive analysis pipeline to evaluate signaling pathway activity based on ligand-receptor interactions and downstream activity (9). Prior to analysis, a differentiation trajectory with known progenitors, maturely differentiated target cells, and neighboring support cells present at the same developmental stage must be defined. This pipeline utilizes the *talklr* package (20) to identify and rank incoming ligand signals to the progenitor cell of interest, filtering for ligand-receptor interactions associated with major signaling pathways of interest. Each interaction is assigned a normalized interaction score, which is calculated by dividing the sum of interaction scores across all pairwise cell-cell interactions. We then utilized the DEsingle package (21) to produce the DEG between the progenitor and maturely differentiated target cells (False Discovery Rate <0.1 and Fold-Change >2). We next used the scMLnet package (22) to generate a multilayer network modeling the upstream ligand–receptor pairs from talklr, downstream transcription factors (TFs), and their target genes from DESingle. Connectivity of each layer of the model was scored to predict which pathway is the most active. Scores were calculated by determining target gene fold-change; mean TF-target genes associated with a given TF; sum of TFs associated with a given receptor; sum of receptors associated with a given ligand; and finally sum of ligands that are associated with a given signaling pathway. Score normalization is performed at each layer. Finally, the pipeline ranks signaling pathways by activity score, indicating the most active pathways including the key drivers of differentiation between progenitor and target maturely differentiated cells (Figure 1A).

#### 2.1.3. Code availability

The custom R codes used in this manuscript are available in https://github.com/Ruohola-Baker-lab/Tooth_sciRNAseq.

### 2.2. Human induced pluripotent stem cell derived odontoblast differentiation guided by Sci-RNA-Seq (iOB)

#### 2.2.1. Human induced pluripotent stem cell culture

Human induced pluripotent stem cell (hiPSC) line WTC-11 (Coriell GM25256) (23, 24) were seeded on 6-well plates and cultured in mTeSR stem cell medium (StemCell Technologies 85850) with daily media changes until cells reach ∼70% confluency (25, 26). Cells were passaged using Accutase (Sigma-Aldrich A6964). ROCK inhibitor (ROCKi) (Stemcell Technologies) is added to mTeSR for initial 24 hours. Cells were passaged every 3 to 4 days until they reached confluency.

#### 2.2.2. hiPSC derived neural crest differentiation (iNC)

This project applies the protocol previously described to produce iPSC derived neural crest (iNC) through dual SMAD inhibition and early WNT activation (27–29). hiPSC are seeded at 32,000 cells per well on 6-well matrigel coated plates and maintained in mTeSR until 70% confluent. Differentiation is induced with addition of Basal Neural Maintenance Media (BNMM), which consists of 250 ml DMEM/F12 + glutamine (Gibco 11320-033) and 250 ml neurobasal media (Gibco 21103-049) supplemented with 2.5 ml N2 (Gibco 17502-048), 5 ml B27 (Gibco 17504-044), 2.5 ml GlutaMax (Gibco 35050-061), 2.5 ml ITS-A (Gibco 51300-044), 400 μl 2-Mercaptoethanol (Thermo Fisher Scientific 21985023), and 2.5 ml NEAA (Thermo Fisher Scientific 11140050). On Day 0, BNMM is supplemented with 10 µM SB 431542 (Biogems BG6675SKU301) and 1 µM LDN 193189 (Biogems BG5537SKU106) for dual SMAD inhibition; inhibition is maintained until Day 4 and Day 3, respectively. On Day 2, WNT is activated via supplementation with 3 µM CHIR 99021 (Tocris Bioscience 4423), which is maintained until Day 11. Media change occurred daily.

#### 2.2.3. Magnetic cell sorting for p75 + iNC

On Day 11, cells were lifted via Accutase (Sigma-Aldrich A6964) and resuspended in an IMAG buffer consisting of 0.5% bovine serum albumin and 2 mM EDTA (Invitrogen 15575-038). iNC were isolated with addition of PE-conjugated p75 (also known as Nerve Growth Factor Receptor, NGFR, and CD271) antibody (Thermo Fisher Scientific 12-9400-42) and Anti-R-PE magnetic beads (BioSciences 557899). p75+ cells were eluted, resuspended in media, and plated on 24-well matrigel coated plates at a density of 250,000 cells per well.

#### 2.2.4. *De novo* designed FGFR1/2c isoform mini binder expression

De novo designed fibroblast growth factor receptor-c (FGFR1/2c) isoform specific mini binder alone (hereby referred to as mb7) or fused to a hexameric scaffold (hereby referred to as C6) were produced as described previously (30–32).

#### 2.2.5. iNC derived odontoblast differentiation

This project applies the DPSC derived to odontoblast protocol previously described by our lab (32), modified for hiPSC and to reflect the full signaling pathway activities as detected by our sci-RNA-seq analysis. In order to fully elucidate the role of FGF signaling in odontoblast development we utilized mb7, which functions as a FGFR antagonist, and C6, which acts as a FGFR agonist (30, 31, 32). p75 + iNC were cultured in Odontogenic Medium, which consists of DMEM + Glutamax (Gibco 10566016), 100 nM dexamethasone (Sigma-Aldrich D4902), 10% fetal bovine serum, 5 mM b-glycerophosphate (Sigma-Aldrich G9422), and 50 µg/ml L-ascorbic acid (Sigma-Aldrich A4544) for 14 days (OB). Odontogenic medium was supplemented with 50 ng/ml BMP4 (Stemcell Technologies 78211) for 7 days followed by 25 ng/ml BMP4 (Stemcell Technologies 78211) and 400 nM SAG (Stemcell Technologies 73412) for 7 days (iOB); 100 ng/ml C6 (30, 31) for 14 days (iOB C6); 100 ng/ml C6 for 7 days followed by 100 ng/ml mb7 (30, 31) for 7 days (iOB C6 to mb7); or 100 ng/ml recombinant basic FGF (Gibco 13256-029) for 14 days (iOB bFGF). All cultures were performed on Matrigel coated plates at a 1:30 dilution and incubated at 37°C and 5% CO_2_ concentration. Each differentiation was performed in triplicate with undifferentiated hiPSC as the negative control.

#### 2.2.6. Protein isolation

Media was aspirated from cell culture plates and the cells were gently rinsed with 1 × PBS. Cells were lysed from 35 mm plates with 131 µl of lysis buffer containing 20 mM Tris–HCl (Sigma-Aldrich 1185-53-1) (pH 7.5), 150 mM NaCl, 15% glycerol (Sigma-Aldrich G5516), 1% triton (Sigma-Aldrich 9002-93-1), 3% SDS (Sigma-Aldrich 151-21-3), 25 mM β-glycerophosphate (Sigma-Aldrich 50020-100G), 50 mM NaF (Sigma-Aldrich 7681-49-4), 10 mM sodium pyrophosphate (Sigma-Aldrich 13472-36-1), 0.5% orthovanadate (Sigma-Aldrich 13721-39-6), 1% PMSF (Roche Life Sciences 329-98-6), 25 U benzonase nuclease (EMD 70664-10KUN), protease inhibitor cocktail (Pierce™ Protease Inhibitor Mini Tablets, Thermo Fisher Scientific A32963), and phosphatase inhibitor cocktail 2 (Sigma-Aldrich P5726), respectively, in a tube. Cell lysate was collected in a fresh Eppendorf tube. 43.33 µl of 4× Laemmli Sample buffer (Bio-Rad 1610747) containing 10% beta-mercaptoethanol (Sigma-Aldrich M7522-100) was added to the cell lysate and then heated at 95°C for 10 min. The boiled samples were either used for Western blot analysis or stored at −80°C.

#### 2.2.7. Western blot assay

Protein samples were thawed via heat block at 95°C for 10 min. A 30 μl of protein sample per well was loaded and separated on a 4%–10% SDS–PAGE gel for 30 min at 250 V. The proteins were then transferred on a nitrocellulose membrane for 12 min using the semi-dry turbo transfer Western blot apparatus (Bio-Rad). Post-transfer, the membrane was blocked in 5% bovine serum albumin (BSA) for 1 h. After 1 h, the membrane was probed with the primary antibodies Nestin (Santa Cruz SC-23927), DSPP (Santa Cruz 7363-2), RUNX2 (Abcam Ab76956) and GAPDH (Cell Signaling Technology 5174S), overnight on a rocker at 4°C. The following day, membranes were washed with 1× TBST 3 × 5-minute washes. The respective HRP-conjugated secondary antibodies (Bio-Rad) were added at 1:10,000 dilution and incubated at room temperature for 1 h. Membranes were then washed with 1× TBST 3 × 5-minute washes. Membranes were then developed using Chemiluminescence and imaged using Bio-Rad ChemiDoc Imager.

#### 2.2.8. Immunofluorescence staining and confocal imaging

Cultured cells were fixed in 4% paraformaldehyde (PFA) then immersed in 1× phosphate-buffered saline (PBS) for 3 × 5-minute washes. Slides were blocked for 60 min at room temperature in a humidified chamber with a blocking buffer consisting of 0.1% Triton X-100% and 5% bovine serum albumin (BSA) (VWR). The primary antibodies DSPP (1:50, Santa Cruz 7363-2), RUNX2 (1:50, Abcam Ab76956), Phalloidin (1:100, Thermo Fisher Scientific A12379) and Vimentin (1:100, Cell Signaling Technology 5741) were incubated overnight at 4°C in a humidified chamber. After 3 × 5-minute washes in PBS in a coplin jar, the slides were transferred to a humidified chamber with secondary antibodies. Secondary antibody goat anti-mouse 488 (1:250, Molecular Probes) was applied for 60 min at room temperature in the same blocking agent. Slides were then rinsed with PBS 4 × 10-min washes in a coplin jar. DAPI (1:50, Molecular Probes) was applied for 10 min at room temperature in PBS. Slides were then rinsed with PBS for 5 min in a coplin jar. Slides were then mounted with Vectashield (Vector Labs) and stored at 4°C until used for imaging. Confocal Imaging was done on a Leica TCS-SPE Confocal microscope using a 40× objective and Leica Software. Images were processed with Fiji software distribution of ImageJ v1.52i (34). Negative controls were performed substituting PBS.

#### 2.2.9. Real-time quantitative reverse transcription-polymerase chain reaction (qPCR)

To analyze gene expression, cells were dissociated and lysed with Trizol (Life Technology) with cell pellets stored at −80°C. RNA purification is performed via TURBO DNA-free™ Kit (Invitrogen) or Aurum™ Total RNA Mini Kit (Bio-Rad), purity and concentration quantification via Nanodrop ND-1000 (Thermo Fisher Scientific). cDNA synthesis via iScript™ cDNA Synthesis Kit (Bio-Rad) or Applied Biosystems™ High-Capacity cDNA Reverse Transcription Kit (Thermo Fisher Scientific), and qPCR performed using oligonucleotide primers for neural crest and odontoblast markers (Table 1), SYBR Green reporter (Applied Biosystems) and 7300 Real-Time PCR System (Applied Biosystems). All qPCR reactions were performed in triplicate, normalized to β-actin and hiPSC, and assessed using the comparative change in threshold cycle (ΔC*_t_*) method.

**Table 1:** qPCR primer sequences.

Cell type	Primer	Forward sequence (5^1^ → 3^1^)	Reverse sequence (5^1^ → 3^1^)
*Endogenous control*	β-ACTIN	TCCCTGGAGAAGAGCTACG	GTAGTTTCGTGGATGCCACA
*Neural crest*	NESTIN	GAAACAGCCATAGAGGGCAA	TGGTTTTCCAGAGTCTTCAGTGA
	PAX3	TGTTTCCCTTTCCTGTGTGG	TTATATCGCCTTGGGCATTG
*Odontoblast*	RUNX2	CATGGCGGGTAACGATGAAA	GTGAAGACGGTTATGGTCAAGG
	DSPP	TGACAGCAATGATGAGAGTG	CACTGGTTGAGTGGTTACTG
	DMP1	GACAGACAAGAAGGAGGAAAC	GCTCTCACTGGTGGTATCT

#### 2.2.10. Statistical analysis

ΔΔC*_t_* values of gene expression of differentiated samples are calculated by normalizing to hiPSC derived odontoblast samples (iOB) and analyzed for significance using Student's *t* test via GraphPad QuickCalcs (www.graphpad.com) for comparisons of two samples or with One-way Anova with Bonferroi's multiple comparison tests for comparison of more than two samples in Prism, GraphPad.

#### 2.2.11. Alizarin red stain mineralization capacity assay

Alizarin red staining (ARS) (Sigma-Aldrich TMS-008) is performed to assess extracellular calcium deposition. Culture medium was aspirated from each well and cells washed with PBS 3×. Cells were fixed in 4% PFA for 15 min at room temperature. PFA was removed and cells washed 3× with diH_2_O. diH_2_O is aspirated off and 1 ml 2% ARS was added per well. Plates were covered in aluminum foil and incubated at room temperature for 45 min with gentle shaking. ARS was removed and cells washed 5× with diH_2_O. Staining was visualized under phase contrast microscopy (Olympus IX70 microscope, Japan). Stain was then released with 10% acetic acid and neutralized with 0.1 M ammonium hydroxide and quantification for OD405 performed via Wallac EnVision system.

## 3. Results

Here we interrogate on a single cell level the intercellular signaling between human odontoblast and ameloblast lineages based on recent fetal tooth germ sci-RNA-seq analysis (9). First, we analyze the signaling pathways involved in human odontoblast development. Second, we dissect the signaling pathways distinguishing two different human tooth types, incisors and molars, in enamel knot and ameloblast development. Third, we utilize the information on the critical signaling pathways involved in human odontoblast development to produce a hiPSC derived odontoblast differentiation protocol (iOB) and dissect the role of FGF signaling using *de nova* designed c-isoform specific FGFR1/2 minibinders (30–32).

### 3.1. Single cell sequencing predicts early FGF and BMP signaling followed by late HH activation as crucial to human odontoblast development

Bioinformatic analysis of the most active signaling pathways during human odontoblast differentiation predicts that fibroblast growth factor (FGF), bone morphogenic protein (BMP), and hedgehog (HH) signaling pathways are critical to directing this developmental trajectory. FGF and BMP are most active during the transition from dental papilla (DP) to preodontoblast (POB) (12–19gw; Figure 1B; Supplementary Figures S1A,H; Supplementary Table S1). BMP activity is reduced by roughly half during the transition from POB to odontoblast (OB) with the majority of signaling derived from HH and Notch pathways (17–22gw; Figure 1B; Supplementary Figures S1A,H; Supplementary Table S3). During the transition from DP to POB, the dental epithelium derived inner enamel epithelium (IEE) is the major source of BMP signaling ligands (Figures 1C,E; Supplementary Table S2), which bind to receptors on the DP (Figure 1G). FGF signaling ligands are most robustly produced by the enamel knot (EK) (Figures 1C,E; Supplementary Table S2) and bind to receptors present on the DP (Figure 1G). Overall, the dental epithelium derived IEE and EK appear to play critical roles in early human odontoblast development, secreting FGF and BMP ligands and inducing DP differentiation to POB in the tooth germ (Figure 1I).

During the transition from POB to OB, the main source of BMP, FGF, and HH signaling ligands is predicted to be the dental epithelium derived pre-ameloblast (PA) (Figures 1D,F; Supplementary Table S4). The receptors for these ligands are highly expressed in the POB (Figure 1H). HH signaling, the most active pathway in the transition, shows significantly increased ligand expression in both the PA and ameloblast (AM) (Figures 1D,F; Supplementary Table S4), which is received by receptors on the POB (Figure 1H). During late odontoblast development it appears that the dental epithelium derived PA is largely responsible for BMP, FGF, and HH ligands secretion, which bind to receptors present on the POB, inducing the transition of human POB to OB (Figure 1J).

### 3.2. Human molar tooth development is delayed compared to incisors

Previous studies have proposed that molars develop more slowly than incisors due to the molar's later eruption date *in vivo* (35). To investigate this, comparative analysis was performed independently on the developing human incisor and molar tooth germ types. The dataset of single cell RNA sequencing of the oral cavity (9) was subset for the dental ectomesenchyme (Supplementary Figures S1B,E) and epithelium lineages (Supplementary Figures S1I,L) by incisor and molar tooth germ type. In the dental ectomesenchyme, incisor and molar subsets showed maintained presence of 6 transcriptionally unique cell types (Supplementary Figures S1B,E,D,G) and 12 transcriptionally unique cell types in the dental epithelium (Supplementary Figures S1I,L,K,N). Pseudotime trajectories were also consistent between incisor and molar tooth germ types in both dental ectomesenchyme (Supplementary Figures S1C,F) and dental epithelium derived tissues (Supplementary Figures S1J,M). Simplified differentiation trajectories illustrate in the dental ectomesenchyme, a common dental ectomesenchyme (DEM) progenitor gives rise to both the DP and the dental follicle (DF). In the odontoblast lineage, DP gives rise to POB, followed by OB. In the dental follicle lineage, the DF gives rise to the subodontoblast (SOB), with a suggested transition of SOB through POB-like state before giving rise to OB (Supplementary Figure S1H). In the dental epithelium, the ameloblast lineage consists of the dental epithelium (DE), which gives rise to the outer enamel epithelium (OEE) followed by the cervical loop (CL), IEE, PA and finally AM. In this study of ameloblast development, CL and IEE cell types were excluded from analysis as molar and incisor tooth germ tissues had been combined prior to single cell RNA sequencing. The enamel knot lineage shares the same progenitor as the ameloblast lineage, with DE giving rise to the enamel knot (EK) (Supplementary Figure S1O).

**Figure 2:**
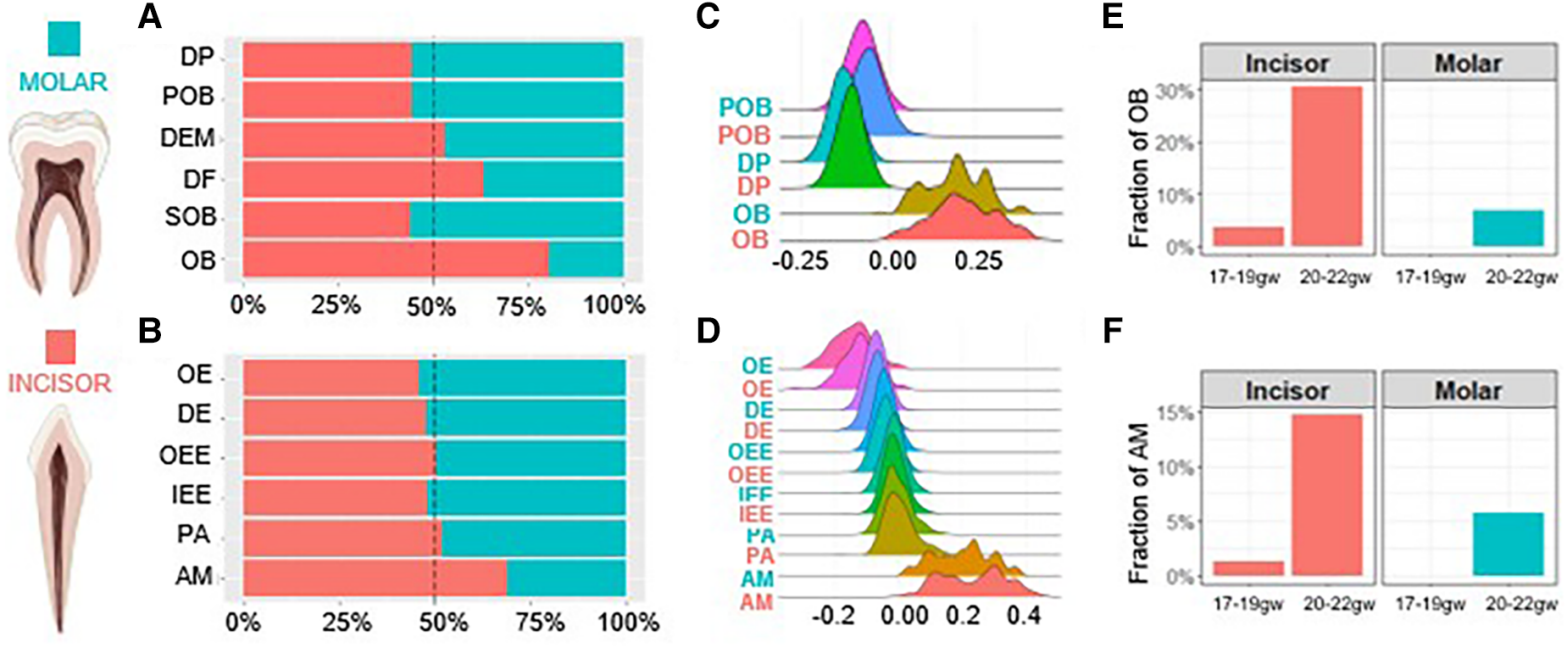
Human molar tooth development is delayed compared to incisors. The proportion of each cell type present within the dental ectomesenchyme (**A**) and dental epithelium (**B**) derived cells. Developmental scores calculated to compare differentiation states in the odontoblast (**C**) and ameloblast (**D**) trajectory. Gestational week at which odontoblasts (**E**) and ameloblasts (**F**) first appear. Incisor (**coral**), molar (**teal**). Graphic generated using Biorender.

The proportion of each cell type present in the developing tooth germ at various developmental stages can define the maturation kinetics. Therefore, we further inspected molar and incisor tooth germ type development on the molecular level by analyzing the proportion of each cell type present in the developing human dental epithelium and dental ectomesenchyme derived tooth tissues. In the dental ectomesenchyme derived tissues, progenitor populations including DP, POB and SOB were richer in the molar while mature DF and OB populations were greater in the incisor. DEM populations were roughly equal (Figure 2A). Similarly, in the dental epithelium derived tissues, progenitor populations OE, DE and IEE were greater in the molar while mature AM populations were denser in the incisor. OEE and PA populations were roughly equal (Figure 2B).

We next compared the differentiation state of each cell type by assigning developmental scores. Scores were calculated by selecting marker genes to determine the maturation (e.g., OB and AM) and progenitor state (e.g., DP and IEE) of each cell type, respectively. The difference between these two sets of scores determines the overall developmental score of each cell type. The results indicate no notable developmental delays between cell types regardless of tissue or tooth germ type (Figures 2C,D). The gestational week (gw) at which OB and AM populations first become present is delayed in molars. While we observe OB and AM at 17–19gw in incisors, the fraction of these mature cells is non-existent in molars before 20–22gw (Figures 2E,F). Together these findings show on a transcriptional level that odontoblasts and ameloblasts develop more rapidly in the incisors compared to the molars, with the molars developing overall at a delayed rate compared to the incisors.

### 3.3. Human enamel knot development is guided by FGF and WNT in the incisor and BMP and ROBO in the molar, with the dental ectomesenchyme as the mutual primary source of signaling ligands

**Figure 3:**
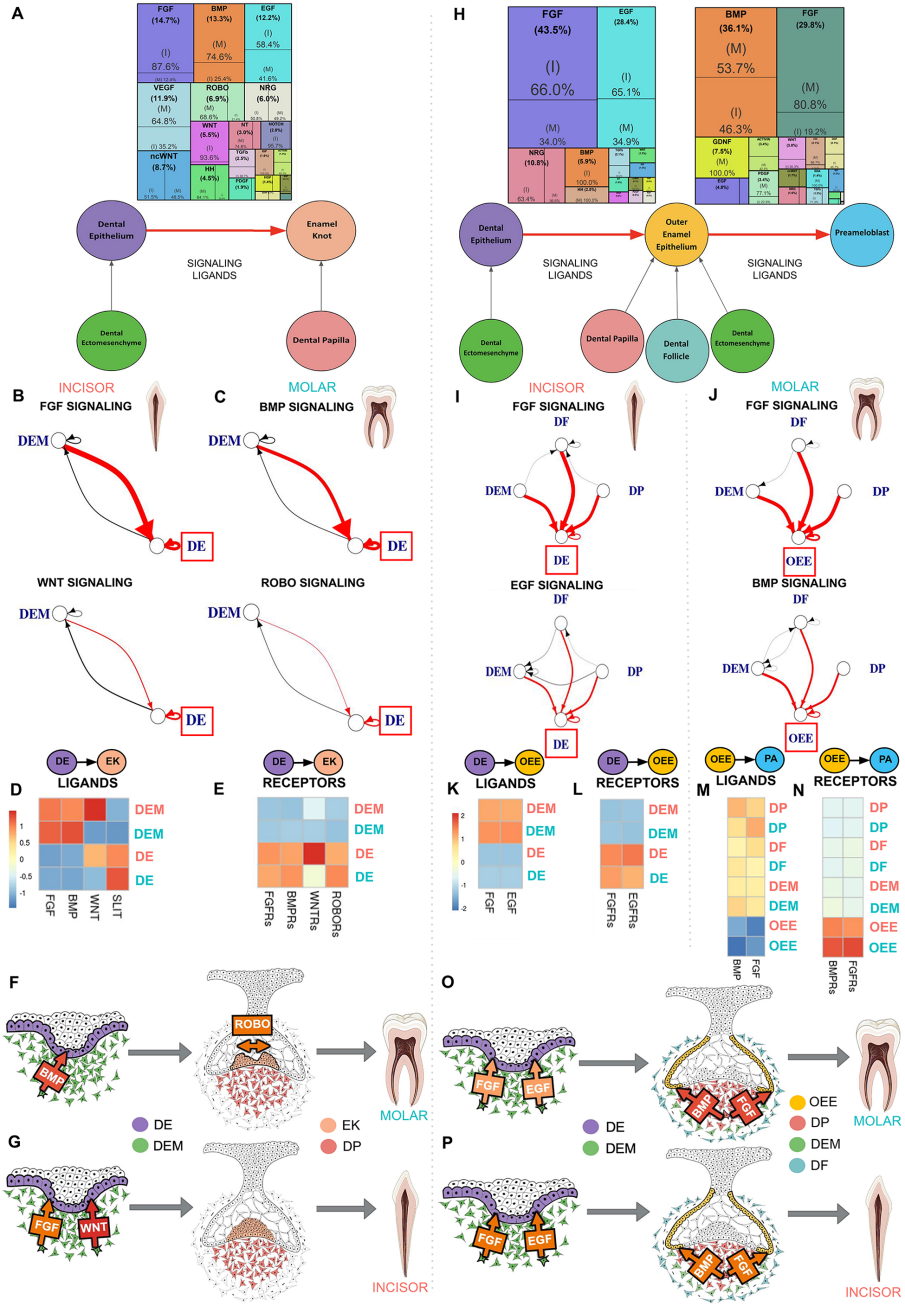
Dental ectomesenchyme derived cells are the primary source of signaling ligands in enamel knot and ameloblast development regardless of tooth type. (**A**) Downstream signaling pathways ranked by activity in the differentiation of the dental epithelium (DE) to enamel knot (EK) segmented by incisor and molar tooth germ type indicates FGF and WNT signaling is critical in the incisor while molars required BMP and ROBO signaling. The sources of critical signaling ligands for the pathways involved in DE to EK transition originate from the dental ectomesenchyme derived tissues in both the incisor (**B**) and molar (**C**). The number of ligand-receptor interactions denoted by the thickness of the line, arrowheads indicating the cell possessing the receptor, and interactions of interest (red) and between support cells (black), with the progenitor of interest in the red box. Heatmaps were generated by aggregating pathway ligand (**D**) and receptor (**E**) gene expression, averaged per cluster. Summary schematics illustrate the dental epithelium and ectomesenchyme derived cells present during the transition from DE to EK. Elevated BMP signaling ligand production by the dental ectomesenchyme (DEM) is observed in the molar, with increased BMPR's on the DE. Increased SLIT ligand and ROBO receptor expression is seen in the molar DE (**F**). Comparatively, in the incisor, FGF and WNT signaling appears to play a critical role in differentiation of DE to EK (**G**). Downstream signaling pathways ranked by activity in the differentiation of the dental epithelium (DE) to outer enamel epithelium (OEE) followed by preameloblast (PA) segmented by incisor or molar tooth germ type indicates incisor tooth germs have increased FGF and EGF signaling during the transition from DE to OEE, while molars require robust FGF and BMP signaling during the maturation of OEE to PA in humans (**H**). The sources of critical signaling ligands for the pathways involved in ameloblast development originate from the dental ectomesenchyme derived tissues in both the incisor and molar in the transition from DE to OEE (**I**) and OEE to PA (**J**). The number of ligand-receptor interactions denoted by the thickness of the line, arrowheads indicating the cell possessing the receptor, and interactions of interest (red) and between support cells (black), with the progenitor of interest in the red box. Heatmaps were generated by aggregating pathway ligand and receptor gene expression, averaged per cluster. During the transition from DE to OEE, the incisor DE is vastly activated by EGF ligands produced by the dental follicle (DF) and FGF ligands produced by the dental papilla (DP) (**K**), which bind to receptors on the DE (**L**). During the transition from OEE to PA, the molar OEE is vastly activated by BMP and FGF ligands produced by the DP (**M**), which bind to receptors on the OEE (**N**). Summary schematics illustrate the dental epithelium and ectomesenchyme derived cells present during the transition from DE to OEE to PA. These bioinformatics based predictions suggest that FGF and EGF signaling is critical for early ameloblast development in the incisor (**P**), while FGF and BMP are required for preameloblast maturation in the molar (**O**), and that the dental ectomesenchyme cells are largely responsible for secretion of the signaling ligands which activate these pathways. The color of arrow denotes expression of signaling ligands and receptors (yellow: mild; orange: moderate; red: high). Incisor (**coral**), molar (**teal**). Graphic generated using InkScape.

Comparative sci-RNA-seq computational analysis of the signaling interactions that guide human enamel knot (EK) development from the dental epithelium (DE) (Figure 3A; Supplementary Figure S1O) predicts several important differences in between incisor and molar tooth germ types. Incisor enamel knot development requires approximately seven times greater FGF signaling and 14 times more WNT signaling than molars. In contrast, molar enamel knot development requires roughly three times greater BMP signaling and two times more ROBO signaling than incisors (Figure 3A; Supplementary Table S5). During the transition from DE to EK, in both incisor and molar tooth germ types, the enamel knot is vastly activated by ligands produced by the dental ectomesenchyme (DEM) (Figures 3B–D; Supplementary Table S6). A notable exception is the ROBO ligand SLIT, which is highly expressed in the DE (Figures 3C,D; Supplementary Table S6). Elevated FGF and WNT ligand production by the DEM (Figures 3B,D; Supplementary Table S6) and FGF receptors (FGFRs) and WNTRs in the DE are observed in the incisor (Figures 3E,G). Comparatively, elevated BMP ligand production by the DEM is observed in the molar (Figures 3C,D; Supplementary Table S6), with increased BMPRs on the DE (Figures 3E,F). Increased SLIT ligand production is seen in the molar DE (Figures 3C,D; Supplementary Table S6), with receptor ROBO expression isolated to the DE (Figures 3E,F), indicating autocrine ROBO signaling within the developing molar enamel knot. These bioinformatics-based predictions suggest BMP and ROBO signaling pathway crosstalk with ROBO ligand SLIT acting as a BMP target. As ROBO signaling is well recognized for guiding axon migration through repulsive action (36), we propose that higher levels of autocrine ROBO signaling within the molar enamel knot plays a similar role, providing repellent patterning for the formation of secondary enamel knots distanced from the site of the primary enamel knot.

### 3.4. Incisor and molar ameloblast development is guided by FGF, EGF and BMP activation with tooth type specific intensity of signaling interactions, with dental ectomesenchyme derived cells act as the shared primary source of signaling ligands

Comparative analysis of the signaling interactions that guide human ameloblast development from dental epithelium (DE) to outer enamel epithelium (OEE) followed by preameloblast (PA) (Figure 3H; Supplementary Figure S1O) predicts several important differences between incisor and molar tooth types. Incisors require approximately two times greater FGF and EGF signaling during the transition from DE to OEE compared to molars (Supplementary Table S7). Molars require elevated BMP and four times greater FGF signaling during the maturation of OEE to PA (Figure 3H; Supplementary Table S9). During the transition from DE to OEE, the incisor DE is vastly activated by EGF ligands produced by the dental ectomesenchyme derived dental follicle (DF) and FGF ligands produced by the dental papilla (DP) (Figures 3I,K,P; Supplementary Table S8), which bind to receptors on the DE (Figures 3l,P). During the transition from OEE to PA, the molar OEE is robustly activated by BMP and FGF ligands produced by the DP (Figures 3J,M,O; Supplementary Table S10), with receptors located on the OEE (Figures 3N,O). These bioinformatics-based predictions suggest that FGF and EGF signaling are critical for early ameloblast development in the incisor (Figure 3P), while increased FGF and BMP activation are required for ameloblast maturation in the molar (Figure 3O). Dental ectomesenchyme cells are largely responsible for secretion of the signaling ligands which activate these pathways (Figures 3O,P).

### 3.5. Early FGF and BMP activation with late FGF agonism using the *de novo* designed FGFR1/2c isoform mini binder C6 and HH activation leads to more mature hiPSC derived odontoblast differentiation *in vitro* (iOB)

**Figure 4:**
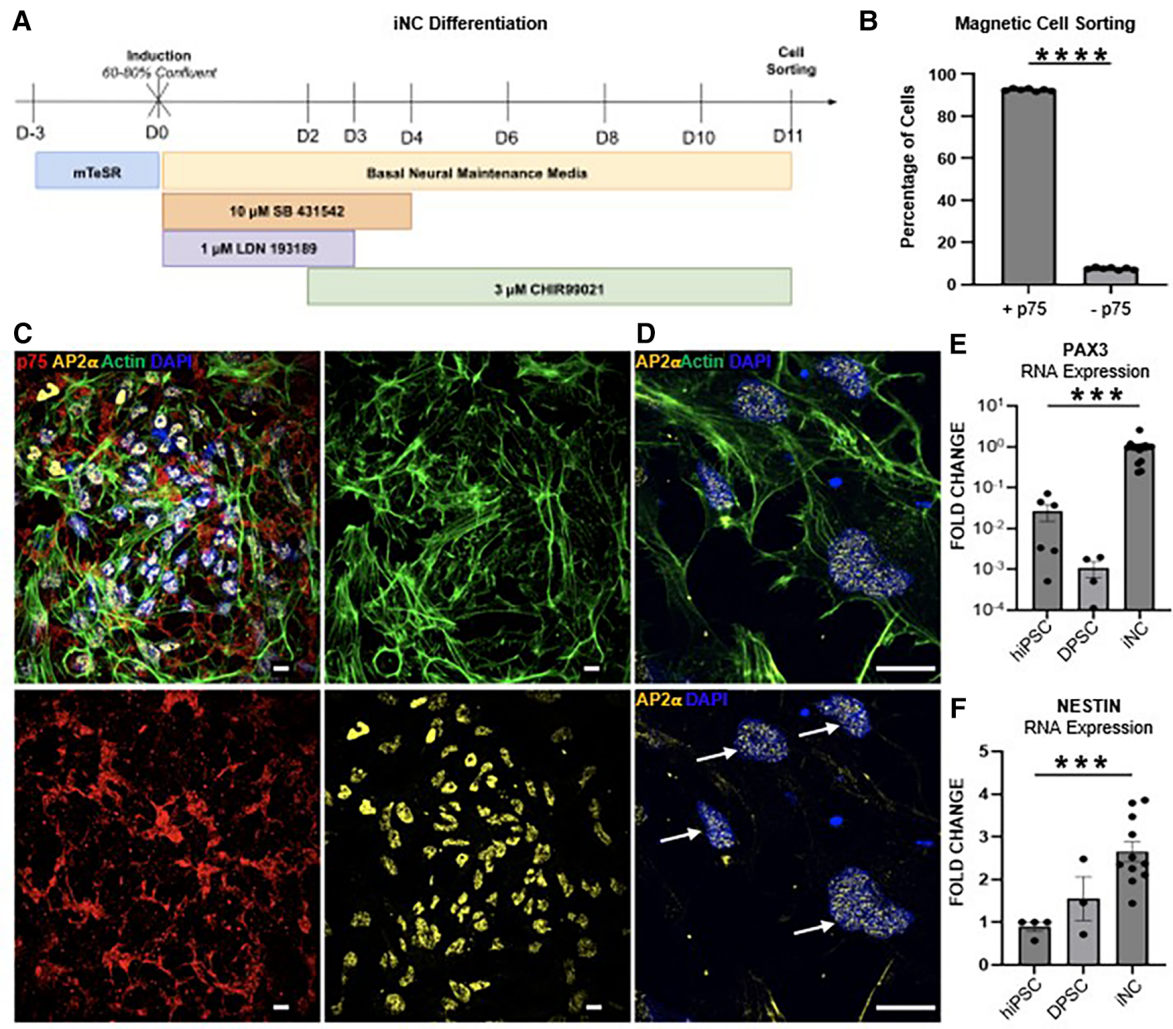
(**A**) Schematic of the 11-day neural crest differentiation protocol (iNC). (**B**) 90% of hiPSC differentiated towards neural crest fate express neural crest marker p75 (CD271) as assessed by magnetic cell sorting. Immunofluorescence staining of iNC show expression of neural crest markers p75 (red) and AP-2ɑ (yellow) (**C**). Scale bar 10 µm. AP-2ɑ (yellow) is localized to the nucleus (white arrows) (**D**). Scale bar 14 µm. (**E**) qPCR of neural crest markers PAX3 and (**F**) NESTIN. Each study was performed in triplicate (*N* = 3), with error bars representing standard error of the mean (SEM). ***p < 0.001; **** *p* < 0.00001.

Using the pathways predicted by single cell analysis of the developing human tooth germ (Figure 1B; Supplementary Figure S1A), we designed a human induced pluripotent stem cell (hiPSC) derived odontoblast differentiation protocol by activating FGF, BMP and HH signaling at appropriate developmental stages. To fully capture the developmental trajectory of the human odontoblast, hiPSC were first differentiated to a neural crest fate (Figure 4A) as described previously (27–29). Successful differentiation was confirmed by magnetic cell sorting for neural crest marker p75, with 90% of cells sorted positively expressing p75 (Figure 4B). Differentiation was further validated by immunofluorescence analysis, which shows induced neural crest cells (iNC) express neural crest markers p75 and transcription factor AP-2ɑ (Figures 4C,D). Expression of neural crest markers NESTIN (37) and PAX3 (38) were next assessed at the transcriptional level via qPCR. We observe a significant 4- and almost 100-fold increase in expression of NESTIN and PAX3, respectively, in iNC compared to undifferentiated hiPSC (Figures 4E,F).

**Figure 5:**
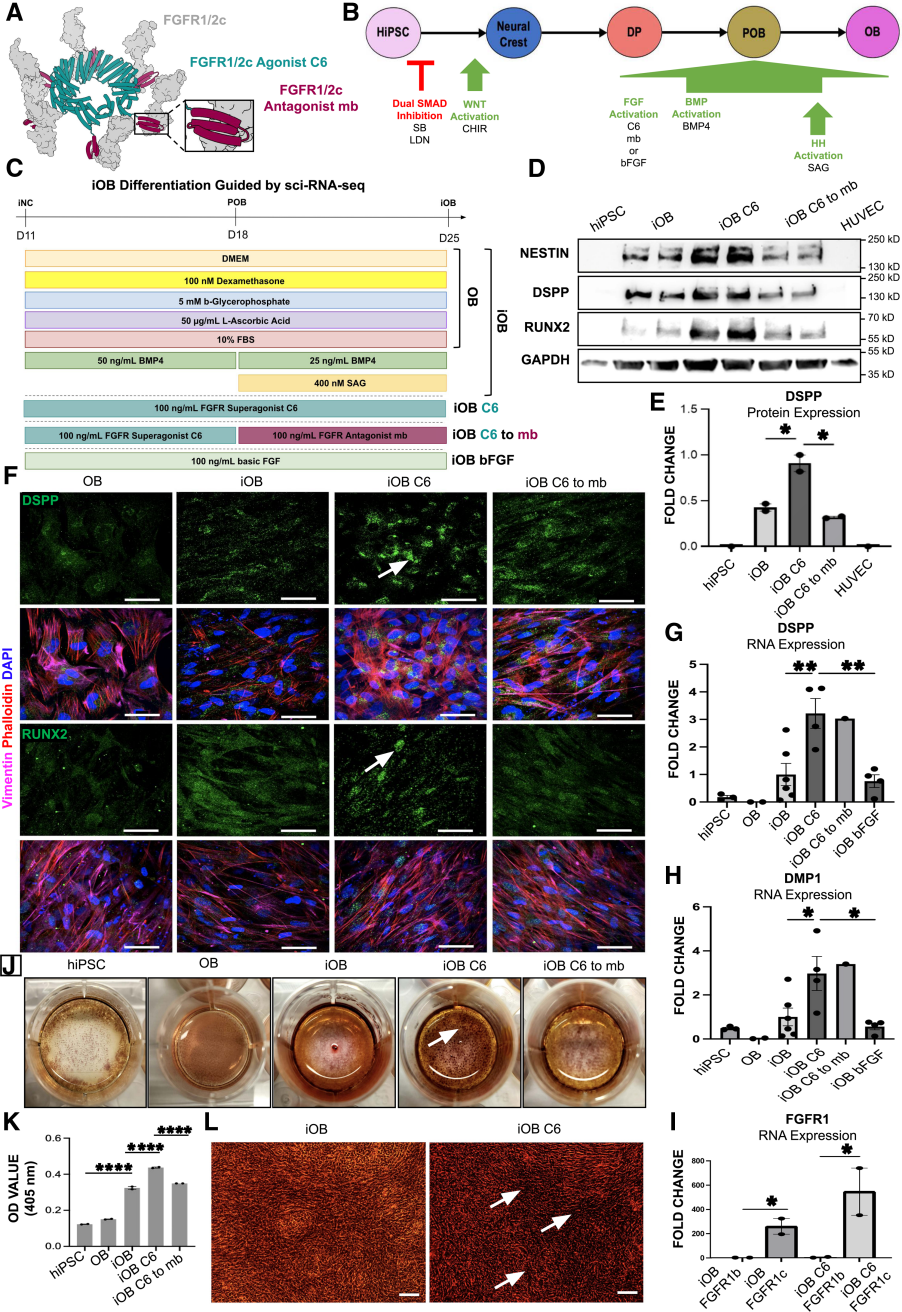
Odontoblast Differentiation Guided by Sci-RNA-Seq Using C6 Produces More Mature Odontoblasts with Increased Mineralization Capacity. (**A**) Model of the de novo designed c-isoform specific FGFR1/2 minibinder (mb7) (maroon) and cyclic, homo-oligomeric, hexameric scaffold fusing six mb7 (C6) (teal) engaging six FGFR1/2c (gray) modified from Edman et al (31). (**B**) 25-day iOB differentiation protocol, which first transitions through iNC before targeting the sci-RNA-seq identified signaling pathways FGF, BMP and HH to produce mature odontoblasts. (**C**) Schematic of the iOB differentiation protocol where iNC are cultured in odontogenic medium (OB); supplemented with BMP4 and SAG (iOB); C6 (iOB C6); C6 followed by mb7 (iOB C6 to mb7); or recombinant basic FGF (iOB bFGF). (**D**) Western blot analysis of NESTIN, RUNX2 and DSPP. (**E**) Quantification of DSPP protein levels. (**F**) Immunofluorescence staining of odontoblast markers DSPP and RUNX2 with white arrows indicating DSPP and RUNX2. Scale bar 50 µm. qPCR analysis of odontoblast markers DSPP (**G**), DMP1 (**H**) and FGFR1c (**I**) expression. Cells stained for​ extracellular calcifications with Alizarin Red Stain (ARS) (**J**). Spectrometric quantification of ARS normalized to hiPSC control (**K**). Higher magnification image of ARS and calcified nodule formation (**L**). Scale bar 20 µm. Each study was performed in triplicate (*N* = 3), with error bars representing standard error of the mean (SEM). *p < 0.05; **p < 0.01; ****p < 0.001.

Next, iNC were biased to an odontoblast fate by culture in odontogenic media as observed in conventional odontoblast differentiation protocols (OB) (32). To activate the BMP and HH signaling pathways identified by computational analysis of sci-RNA-seq data (Figure 1B; Supplementary Figure S1A), odontogenic media was supplemented with BMP ligand BMP4 and HH pathway agonist SAG (iOB). In order to elucidate the role of FGF signaling in odontoblast differentiation, as predicted by sci-RNa-seq analysis, odontogenic medium was additionally supplemented with C6 (iOB C6); C6 followed by mb7 (iOB C6 to mb7) (30–33) (Figure 5A); or basic FGF (iOB bFGF) (Figures 5B,C).

hiPSC derived odontoblast cells (iOB) have increased expression of mature odontoblast markers DSPP (10) and RUNX2 (39) at the protein level as assessed by Western Blot (Figure 5D). Further, iOB treated with C6 (iOB C6) show a significant two-fold increase in DSPP expression compared to both iOB and iOB C6 to mb7 cells (Figures 5D,E). No significant change in RUNX2 or NESTIN was observed at the protein level (Figure 5D; Supplementary Figures S2A,B). Successful differentiation of iNC to an odontoblast fate was further validated by immunofluorescence analysis, which shows iOB C6 cells most strongly express odontoblast markers DSPP and RUNX2 compared to OB, iOB, and iOB C6 to mb7 (Figure 5F). Expression of odontoblast markers RUNX2, DSPP, and DMP1 were next assessed at the transcriptional level via qPCR, which indicates significantly greater expression of both mature odontoblast markers DSPP and DMP1 in iOB C6. Compared to iOB and iOB bFGF, iOB C6 cells show 3- and 5-fold increases in expression of DSPP and DMP1, respectively (Figures 5G,H). No significant changes in RUNX2 expression were observed (Supplementary Figure S2C). The *de novo* designed mini binders interact exclusively with the FGFR1/2c isoform, allowing differential functional analysis of the FGFR1/2c- and b-isoforms (30, 31). We observe significantly increased FGFR1c expression in iOB and iOB C6 treated cells (Figure 5I). Lastly, biomineralization capacity was assessed via Alizarin Red Staining. iOB C6 shows significantly enhanced deposition of mineralized matrix compared to iOB as evidenced by increased mineralized nodule formation, while iOB C6 to mb7 show a significant decrease in biomineralization capacity compared to iOB C6 (Figures 5J–L; Supplementary Figures S2D–H).

## 4. Discussion

### 4.1. Roundabout signaling predicted to shape molar enamel knot formation

Tooth development requires continual, reciprocal signaling between the dental epithelium and dental ectomesenchyme derived tissues (5). Isolated tooth epithelium or dental ectomesenchyme do not result in tooth formation (6). Odontogenic potential shifts from dental epithelium to dental ectomesenchyme at the cap stage of tooth development (11). However, whether determination of tooth type (e.g., if an incisor or molar will form) lies with the dental ectomesenchyme or dental epithelium remains unknown. Previous studies show the enamel knot is a critical receiving cell for tooth type determination, triggering proliferation of neighboring dental ectomesenchyme cells and epithelium derived cervical loop cells (12). We sought to reveal the signaling determinants of enamel knot formation in the incisor compared to the molar. This analysis indicates WNT and FGF are the most active signaling pathways in incisor enamel knot development, with ligand secretion largely from the dental ectomesenchyme. FGF4 and SLIT1 are currently the best biomarkers for the developing murine enamel knot, as they are the sole genes observed to be expressed in both primary and secondary enamel knots (40, 41). We have previously shown localized FGF4 expression in the human incisor enamel knot (9), indicating a shared expression pattern between murine and human enamel knot development. While FGF4 is known to stimulate cusp growth by inducing proliferation of dental epithelium and ectomesenchyme derived cells, the role of SLIT/ROBO signaling in enamel knot development is not fully understood.

Molar enamel knot formation is predicted to be guided by BMP ligand production by the neighboring dental ectomesenchyme, followed by ROBO activation in the dental epithelium. BMP-SLIT crosstalk has been observed in myoblasts and fibroblasts (42). We hypothesize that the ROBO ligand SLIT is a BMP target in the dental epithelium, activation of which results in increased ROBO/SLIT activity in molar enamel knot development. SLIT proteins have an evolutionarily conserved role in axon guidance as repulsive ligands for ROBO receptors and are best known for mediating axon migration (36). We propose that the increased expression of SLIT ligand and ROBO receptor in molar enamel knot development acts in a similar chemorepulsive fashion, inducing migration of the dental epithelium cells that will give rise to secondary enamel knots, resulting in multiple cusp formation observed in molars. While the cell fate trajectories of primary and secondary enamel knots are not fully understood (43, 44), this study illuminates that ROBO/SLIT signaling may play a critical role in molar enamel knot patterning.

### 4.2. Human ameloblast development relies on dental ectomesenchyme produced signaling ligands

Ameloblast development has been shown to be reliant upon signaling ligands produced by the dental ectomesenchyme derived cells (9). However, the specific cells of the dental pulp and how their signaling interactions with the neighboring dental epithelium derived cells impact ameloblast development remained unknown. Here we predict that both incisor and molar ameloblasts require FGF, EGF, and BMP signaling during development. FGF and EGF signaling appear critical for early ameloblast development in the incisor, while FGF and BMP are suggested for ameloblast maturation in the molar. The dental ectomesenchyme cells are largely responsible for secretion of the signaling ligands that activate these pathways in both tooth germ types. Crosstalk between BMP and FGF signaling pathways have been shown to influence the site of murine tooth formation, regulating areas of cell proliferation and apoptosis (45). This supports a role for BMP and FGF crosstalk in human ameloblast development, with signaling ligands originating from the dental ectomesenchyme derived cells.

### 4.3. The need for hiPSC derived odontoblasts is paramount for regenerative therapies and disease modeling

Odontoblasts are responsible for the formation of the tooth's dentin, which composes most of the tooth's mineralized tissue. Dentin provides the tooth's toughness, or resistance to crack propagation, and tensile strength, or distribution of biomechanical forces to the surrounding periodontium. While odontoblasts persist throughout life and can respond to injury by secreting tertiary dentin, their number and ability to produce dentin significantly decreases with age, posing a challenge to regenerative dentistry. If the primary odontoblasts are lost, dental pulp stem cells (DPSC) are induced to differentiate into odontoblast-like cells, forming reparative dentin (46). DPSC have previously been shown to successfully differentiate towards osteogenic and odontogenic fates (46–48) and have been characterized by our lab in detail (32). However, DPSC expansion and regeneration capacity is limited (49), showing a dramatic decrease in regenerative capacity with increased age (50).

As the need for odontoblast regeneration is critical, previous studies have explored stem cell derived odontoblast differentiation protocols. In animal models, odontoblast-like cells have been produced from murine iPSC (miPSC) through co-culture with dental epithelium, with the goal of mimicking early tooth development in which the odontoblasts are in proximity with the ameloblasts (51, 52); and through gene transfection of miPSC to increase BMP4 and PAX9 expression (53). These methods are not ideal for therapeutic application, as access to developing human oral epithelium is limited and human gene therapy requires further study of off-target effects before being clinically practical. Interestingly, a recent study found supplementation of miPSC derived neural crest-like cells with BMP4, FGF8, and WNT3a increases expression of odontoblast marker genes and odontoblast-like morphology (54), supporting a vital role for these signaling pathways in odontoblast maturity. In humans, BMP4 supplementation has been found to produce more mature iPSC derived odontoblast-like cells (55). However, this method did not transition through a neural crest state prior to odontoblast differentiation, leaving a gap missing a crucial stage in odontoblast formation and preventing full analysis of odontoblast development needed for regenerative therapies and disease modeling.

### 4.4. A first-of-its-kind insight into the fate drivers of human odontoblasts

Our studies have revealed, for the first time at the single cell level, the signaling pathways that govern each transition between odontoblast cell lineage identities. Previous studies of hypodontia and tooth agenesis have shown that disruption of FGF, BMP, and HH signals result in defective tooth development. However, the detail with which our study has revealed the role of these pathways at various points in odontoblast development may more mechanistically explain how defects in these pathways lead to tooth loss or tooth agenesis. Our computational analysis identified FGF, BMP, and HH signaling to play critical roles in human odontoblast development, with the majority of signaling ligands secreted by neighboring dental epithelium tissues. Odontoblasts are believed to develop through reciprocal, repeated signaling interactions with the dental epithelium derived ameloblasts. Our signaling pathway analysis indicates that the majority of signaling ligands critical for odontoblast development are produced by the dental epithelium derived inner enamel epithelium and pre-ameloblast at early and late tooth development, respectively. Interestingly, as the POB transitions to OB, the bulk of BMP signaling ligands received are secreted by the SOB, indicating a supportive role for this novel cell type in human OB development. While previous studies have focused on the role of a single signaling pathway, many others have highlighted the importance of crosstalk between pathways in tooth development and maintenance (56–58). Our predictive pathway analysis highlights not only the primary pathway responsible for each stage, but ranks the other pathways involved, meaning that our study will facilitate the investigation into both previously identified and yet undescribed crosstalk in driving forward development. This analysis will facilitate more detailed and informed studies on degenerative dental diseases and can lead to the development of more effective ways to mitigate or reverse tooth loss. This knowledge can be used to develop therapeutic agents to induce dentinogenesis clinically and was applied here to develop an efficient hiPSC derived odontoblast differentiation protocol (iOB).

### 4.5. Single cell RNA sequencing guided targeting of FGFR1 C-isoform using *de novo* mini binders produces more mature hiPSC derived odontoblasts *in vitro* (iOB C6)

Analyzing the signaling interactions that guide human odontoblast development allowed us to predict the signaling molecules needed to recapitulate odontoblast differentiation *in vitro*. Importantly, single cell analysis of the odontoblast lineage indicated that BMP, HH, and FGF signaling are critical to human odontoblast development. We found that iNC cultured in odontogenic medium will differentiate towards an odontoblast fate (OB). Activation of sci-RNA-seq detected signaling pathways BMP, HH, and FGF via supplementation with BMP4, SAG, and without (iOB) or with bFGF (iOB bFGF) produces more mature odontoblast cells illustrating increased expression of odontoblast markers DSPP and DMP1 and increased mineralization capacity. Agonism of c-isoform FGFR1/2 using C6 produced the most advanced odontoblasts with significantly increased expression of mature odontoblast markers DSPP and DMP1 at both the RNA and protein levels, with significantly enhanced mineralization capacity (iOB C6). These findings indicate that while sci-RNA-seq identified BMP and HH signaling play critical roles in early human odontoblast development, it is the agonist of FGF signaling using the *de novo* designed c-isoform specific FGFR1/2 hexameric minibinder C6 that produces odontoblasts with significantly greater maturation and biomineralization capacity, loss of which results in inhibited mineral deposition activity.

A limitation of single cell RNA sequencing is the insensitivity to splice variants of a given signaling pathway, grouping all isoforms of signaling ligands and receptors under the large umbrella of the overall signaling pathway (9). FGFR1 is known to exist as two alternatively spliced variants, the b- and c-isoforms (59), which are thought to play unique roles in development. To further elucidate the role of FGFR1 splice variants in human odontoblast development, we utilized the *de novo* designed mini binder (referred to as mb7) which binds the FGFR1/2c isoform with high specificity (30, 31). Previous studies have shown that clustering FGFR1/2c by directly fusing mb7 to a cyclic, homo-oligomeric, hexameric scaffold (referred to as C6) generates FGF signaling pathway agonism targeting the FGFR1/2c isoform exclusively (30, 31). We exposed iNC to odontogenic medium containing equivalent concentrations of bFGF, which indiscriminately activates both FGFR1/2c and FGFR1/2b, and C6. We found that while iOB bFGF cells show increased DSPP and DMP1 expression compared to cells treated with a conventional odontoblast differentiation method (OB), iOB C6 cells have significantly higher expression of these mature odontoblast markers compared to iOB bFGF, in addition to more robust mineralization capacity indicated by greater mineralized nodule formation. Intriguingly, iOB C6 cells show high expression of FGFR1c compared to FGFR1b, indicating that FGFR1c is the prevalent isoform in odontoblasts and supporting C6's previously reported role as a FGF signaling pathway agonist (30, 31). Thus, our findings suggest that FGFR1c is upregulated in functional odontoblasts and specifically plays a crucial role in driving odontoblast maturity (60–62) rather than odontoblast progenitor proliferation (63).

### 4.6. iOB impact on disease modeling

The hiPSC derived odontoblast differentiation protocol guided by single cell RNA sequencing utilizing the *de novo* designed FGFR1/2c mini binder C6 now reveals a novel, highly simplified method to identify the precise signaling pathways required during the stages of human odontoblast development. The method described in this study, using the *de novo* mini binders to unravel the FGF signaling required for odontoblast maturation in humans, will be generally applicable and specific to any signaling pathway analyzed in the differentiation of normal and disease organoids. This finding implies great potential for *de novo* designed mini binders as therapeutic agents to induce odontoblast differentiation in clinical cases of pulp exposure or deep caries, as well as generation of mature iOB to be used for tooth organoid generation. Beyond bioengineering lost dentin tooth structure, a method of producing functional odontoblasts from hiPSC serves as a model essential to studying genetic diseases affecting dentin formation. This includes Tricho-Dento-Osseous (TDO) syndrome, a rare but highly penetrant autosomal dominant disorder associated with mutations in the homeodomain transcription factor DLX3 (64), producing debilitating dental defects leading to increased incidence of dental caries, tooth fracture, pulpal necrosis, and tooth loss. In order to develop TDO therapies, it is critical to deepen our understanding of DLX3's role in human odontoblast development.

## 5. Conclusion

Here we provide unprecedented insights at the single cell level into the signaling interactions guiding human odontoblast and ameloblast development, as well as those that determine incisor and molar tooth type identity. We propose a novel role for ROBO chemorepulsive signaling in molar enamel knot formation and share knowledge of the signaling patterns that guide enamel knot development in the incisor and molar, allowing specification of cusp formation and crown morphology in forthcoming organoid studies. Analysis of the signaling patterns predicted from sci-RNA-seq of the developing human tooth germ generates a hiPSC derived odontoblast differentiation method utilizing the *de novo* designed FGFR1/2c mini binder (iOB C6). This study marks the first application of *de novo* designed proteins in the field of regenerative dentistry and provides a profound tool to be used for therapeutic and disease modeling goals. Our findings support a functional role for FGFR1c isoform in human odontoblast maturation. Future co-culture studies of iOB C6 with our previously described hiPSC derived ameloblasts (iAM) (9) will allow further dissection of the signaling patterns exchanged between dental epithelium and dental ectomesenchyme derived tissue types during tooth development, likely driving advanced maturation of both odontoblast and ameloblast cell types.

## Data Availability

The data presented in the study are deposited in the GEO Database repository, accession number GSE184749. The custom R codes used in this manuscript are available in https://github.com/Ruohola-Baker-lab/Tooth_sciRNAseq.

## Supplementary material

Supplementary Figure S1 A Single Cell Atlas of the Developing Human Incisor and Molar Dental Cell Types. (A) Downstream signaling pathways ranked by activity with detailed signaling ligands per pathway in odontoblast development indicate FGF and BMP are critical to the dental papilla (DP) as it transitions to preodontoblast (POB); HH, BMP, and NOTCH are the most active as POB transitions to odontoblast (OB). UMAP graph of subclustered incisor (B) and molar (E) tooth germ type dental mesenchyme derived cells from the total dataset identified conserved 6 transcriptionally unique clusters identified by collating highly expressed cluster-specific genes including dental papilla (DP), preodontoblast (POB), odontoblast (OB), subodontoblast (SOB), odontoblast (OB), dental ectomesenchyme (DEM), and dental follicle (DF). Pseudotime trajectory analysis for dental mesenchyme derived cells suggest two progenitors DP and DEM (blue), that give rise to differentiated OB (yellow) for both incisor (C) and molar (F). Heatmaps for putative marker genes for each dental mesenchyme cell type were produced for incisor (D) and molar (G). Simplified differentiation trajectory tree illustrating a common DEM progenitor gives rise to both DP and DF. In the OB lineage (red), DP gives rise to POB, followed by OB; DF lineage (grey) indicates DEM giving rise to DF, which gives rise to SOB, with a suggested transition through POB-like state before giving rise to OB (H). UMAP graph of subclustered incisor (I) and molar (L) tooth germ type dental epithelium derived cells from the total dataset yielded 12 unique clusters that we identified by collating highly expressed cluster-specific genes including: oral epithelium (OE), dental epithelium (DE), enamel knot (EK), inner and outer enamel epithelium (IEE, OEE), cervical loop (CL), inner and outer stratum intermedium (SII, SIO), inner and outer stellate reticulum (SRI, SRO), pre-ameloblasts (PA) and ameloblast (AM). Pseudotime trajectory analysis for dental epithelium derived cells suggests the OE directly gives rise to DE, followed by EK. The DE also gives rise to the SR and OEE lineages, which give rise to SI, IEE/PA, and AM in both the incisor (J) and molar (M). Heatmaps for putative marker genes for each dental epithelium cell type were produced for both incisor (K) and molar (N). Simplified differentiation trajectory tree illustrating a common DE progenitor gives rise to EK (gray), SR (gray) and OEE lineages (red) (O) (9).

Supplementary Figure S2 C-Isoform Specific Activation of FGFR1 with *De Novo* Designed Mini Binder C6 Promotes Improved Mineralization Capacity of iOB. Quantification of Western Blot protein level of RUNX2 (A) and NESTIN (B). qPCR analysis of odontoblast marker RUNX2 (C). Extracellular calcifications assessed via Alizarin Red Stain (ARS) in iNC cultured in odontogenic medium (OB) (D); supplemented with BMP4 and SAG (iOB) (E); C6 (iOB C6) (F); C6 followed by mb7 (iOB C6 to mb7) (G); or undifferentiated hiPSC (H). Scale bar 20 µm.
