# Corrigendum: Single cell RNA sequencing reveals human tooth type identity and guides *in vitro* hiPSC derived odontoblast differentiation (iOB)

**DOI:** 10.3389/fdmed.2023.1278809

**Published:** 2023-11-06

**Authors:** Sesha Hanson-Drury, Anjali P. Patni, Deborah L. Lee, Ammar Alghadeer, Yan Ting Zhao, Devon Duron Ehnes, Vivian N. Vo, Sydney Y. Kim, Druthi Jithendra, Ashish Phal, Natasha I. Edman, Thomas Schlichthaerle, David Baker, Jessica E. Young, Julie Mathieu, Hannele Ruohola-Baker

**Affiliations:** ^1^ Department of Oral Health Sciences, School of Dentistry, University of Washington, Seattle, WA, United States; ^2^ Department of Biochemistry, School of Medicine, University of Washington, Seattle, WA, United States; ^3^ Institute for Stem Cell and Regenerative Medicine, School of Medicine, University of Washington, Seattle, WA, United States; ^4^ Department of Genetic Engineering, SRM Institute of Science and Technology, Chennai, India; ^5^ Department of Biomedical Dental Sciences, College of Dentistry, Imam Abdulrahman bin Faisal University, Dammam, Saudi Arabia; ^6^ Department of Biology, University of Washington, Seattle, WA, United States; ^7^ Department of Biotechnology, SRM Institute of Science and Technology, Chennai, India; ^8^ Department of Bioengineering, University of Washington, Seattle, WA, United States; ^9^ Institute for Protein Design, University of Washington, Seattle, WA, United States; ^10^ Molecular and Cellular Biology Graduate Program, University of Washington, Seattle, WA, United States; ^11^ Medical Scientist Training Program, University of Washington, Seattle, WA, United States; ^12^ Department of Laboratory Medicine and Pathology, University of Washington, Seattle, WA, United States; ^13^ Department of Comparative Medicine, School of Medicine, University of Washington, Seattle, WA, United States

**Keywords:** odontoblast, single cell RNA sequencing, enamel knot, cell signaling, *de novo* designed mini protein binders, human tooth, regenerative dentistry, tooth development

A Corrigendum on Single cell RNA sequencing reveals human tooth type identity and guides *in vitro* hiPSC derived odontoblast differentiation (iOB) By Hanson-Drury S, Patni AP, Lee DL, Alghadeer A, Zhao YT, Ehnes DD, Vo VN, Kim SY, Jithendra D, Phal A, Edman NI, Schlichthaerle T, Baker D, Young JE, Mathieu J and Ruohola-Baker H (2023) Single cell RNA sequencing reveals human tooth type identity and guides *in vitro* hiPSC derived odontoblast differentiation (iOB). Front. Dent. Med 4:1209503. doi: 10.3389/fdmed.2023.1209503

**Error in Author List**

In the published article, there was an error in the **author list**, and authors Natasha I. Edman and Thomas Schlichthaerle were erroneously excluded. The corrected **author list** and **affiliations** appear below:

Sesha Hanson-Drury^1,2,3^, Anjali P. Patni^1,2,3,4^, Deborah L. Lee^1,2,3^, Ammar Alghadeer^1,2,3,5^, Yan Ting Zhao^1,2,3^, Devon Duron Ehnes^1,2,3^, Vivian N. Vo^2,3,6^, Sydney Y. Kim^1,3^, Druthi Jithendra^3,7^, Ashish Phal^2,3,8^, Natasha I. Edman^2,9,10,11^, Thomas Schlichthaerle^2,9^, David Baker^2,3,9^, Jessica E. Young^3,12^, Julie Mathieu^3,13^ and Hannele Ruohola-Baker^1,2,3,6,8*^

^1^Department of Oral Health Sciences, School of Dentistry, University of Washington, Seattle, WA, United States

^2^Department of Biochemistry, School of Medicine, University of Washington, Seattle, WA, United States

^3^Institute for Stem Cell and Regenerative Medicine, School of Medicine, University of Washington, Seattle, WA, United States

^4^Department of Genetic Engineering, SRM Institute of Science and Technology, Chennai, India

^5^Department of Biomedical Dental Sciences, College of Dentistry, Imam Abdulrahman bin Faisal University, Dammam, Saudi Arabia

^6^Department of Biology, University of Washington, Seattle, WA, United States

^7^Department of Biotechnology, SRM Institute of Science and Technology, Chennai, India,

^8^Department of Bioengineering, University of Washington, Seattle, WA, United States

^9^Institute for Protein Design, University of Washington, Seattle, WA, United States

^10^Molecular and Cellular Biology Graduate Program, University of Washington, Seattle, WA 98195, USA

^11^Medical Scientist Training Program, University of Washington, Seattle, WA 98195, USA

^12^Department of Laboratory Medicine and Pathology, University of Washington, Seattle, WA, United States

^13^Department of Comparative Medicine, School of Medicine, University of Washington, Seattle, WA, United States

**Missing Citation**

In the published article Park JS, Choi J, Cao L, Mohanty J, Suzuki Y, Park A, Baker D, Schlessinger J, Lee S. Isoform-specific inhibition of FGFR signalizing achieved by a de-novo-designed mini-protein. Cell Reports. (2022) 41(4):111545. doi: 10.1016/j.celrep.2022.111545. was not cited in the article. The citation has now been inserted in **Materials and methods**, *De novo designed FGFR1/2c isoform mini binder expression*, paragraph 1 and should read:

“De novo designed fibroblast growth factor receptor-c (FGFR1/2c) isoform specific mini binder alone (hereby referred to as mb7) or fused to a hexameric scaffold (hereby referred to as C6) were produced as described previously (30, 31, 32).”

**Error in Figure/Table**

In the published article, there was an error in **Figure 5** **caption** in which Edman NI, Redler RL, Phal A, Schlichthaerle T, Srivatsan SR, Etemadi A, et al. Modulation of FGF pathway signaling and vascular differentiation using designed oligomeric assemblies. bioRxiv. [preprint]. (2023). doi: 10.1101/2023.03.14.532666 was not cited. The corrected **Figure 5 caption** appears below.

Odontoblast Differentiation Guided by Sci-RNA-Seq Using C6 Produces More Mature Odontoblasts with Increased Mineralization Capacity. (A) Model of the *de novo* designed c-isoform specific FGFR1/2 minibinder (mb7) (maroon) and cyclic, homo-oligomeric, hexameric scaffold fusing six mb7 (C6) (teal) engaging six FGFR1/2c (gray) modified from Edman et al. (31). (B) 25-day iOB differentiation protocol, which first transitions through iNC before targeting the sci-RNA-seq identified signaling pathways FGF, BMP and HH to produce mature odontoblasts. (C) Schematic of the iOB differentiation protocol where iNC are cultured in odontogenic medium (OB); supplemented with BMP4 and SAG (iOB); C6 (iOB C6); C6 followed by mb7 (iOB C6 to mb7); or recombinant basic FGF (iOB bFGF). (D) Western blot analysis of NESTIN, RUNX2 and DSPP. (E) Quantification of DSPP protein levels. (F) Immunofluorescence staining of odontoblast markers DSPP and RUNX2 with white arrows indicating DSPP and RUNX2. Scale bar 50 µm. qPCR analysis of odontoblast markers DSPP (G), DMP1 (H) and FGFR1c (I) expression. Cells stained for extracellular calcifications with Alizarin Red Stain (ARS) (J). Spectrometric quantification of ARS normalized to hiPSC control (K). Higher magnification image of ARS and calcified nodule formation (L). Scale bar 20 µm. Each study was performed in triplicate (*N* = 3), with error bars representing standard error of the mean (SEM). *p < 0.05; ** *p* < 0.01; **** *p* < 0.001.

**Incorrect Funding**

In the published article, there was an error in the **Funding** statement. The funding for author Thomas Schlichthaerle was omitted. The correct **Funding** statement appears below.

This work is supported by grants from the National Institutes of Health 1P01GM081619, R01GM097372, R01GM97372-03S1 and R01GM083867 and the NHLBI Progenitor Cell Biology Consortium (U01HL099997; UO1HL099993) for HRB. Additional support provided by the BBI grants for JM and HRB. This work was also supported by Dr. Douglass L. Morell Research Fund for HRB, AA, and YTZ. The Birth Defects Research Laboratory was supported by NIH award number 5R24HD000836, to IAG, from the Eunice Kennedy Shriver National Institute of Child Health and Human Development. TS was supported by the European Molecular Biology Organization via ALTF191-2021. SHD, YTZ and DE were supported by National Institutes of Health T90DE021984. SHD was supported by ARCS. AA was supported by Imam Abdulrahman bin Faisal University and the Saudi Arabian Cultural Mission (SACM).

**Incorrect Acknowledgements**

In the published article, there was an error in the **Acknowledgements** statement. The Institute for Protein Design Core was omitted. The correct **Acknowledgements** statement appears below.

We thank the Ruohola-Baker lab for discussions that propelled this work in new directions. Infencia Xavier for her technical help. Chizuru Kinoshita for assistance culturing and sorting neural crest cells. Jennifer Dempsey, Ian Phelps, Dr. Diana O'Day from BDRL for processing/collecting samples and nuclei isolation. Cailyn Suprrell, Aishwarya Gogate and Lea Starita from BBI for technical support and guidance. The Institute for Protein Design Core staff for assistance in protein production.

**Incorrect Author Contributions**

In the published article, there was an error in the **Author Contributions** statement. Authors NIE and TS were omitted. The correct **Author Contributions** statement appears below.

SH-D, AA and HR-B conceptualized the research. SH-D, DL, AA and DE conducted bioinformatic analysis. SH-D, APP, VV, SK, DJ and YZ performed *in vitro* experiments. NIE, TS, AP and DB contributed the *de novo* designed FGFR1/2c mini binders. SH-D, DL, DE, JM, SK and HR-B contributed to writing. JM, JY and HR-B supervised the study. All authors contributed to the article and approved the submitted version.

**Text Corrections**

In the published article, there was an error. The methodology used to design the *de novo* FGFR1/2 minibinders and their c-isoform specificity was not clarified.

A correction has been made to **Abstract**. This sentence previously stated:

“Further, we elucidate the critical role of FGF signaling in odontoblast maturation and biomineralization capacity using the *de novo* designed FGFR1/2c isoform minibinder scaffold C6.”

The corrected sentence appears below:

“Further, we elucidate the critical role of FGF signaling in odontoblast maturation and its biomineralization capacity using the *de novo* designed FGFR1/2c isoform specific minibinder scaffolded as a C6 oligomer that acts as a pathway agonist.”

A correction has been made to **Introduction**, Paragraph 5. This sentence previously stated:

“Further,we apply the sci-RNA-seq predicted signaling pathways to generate a hiPSC derived odontoblast differentiation method (iOB) using *de novo* AI designed FGFR1/2c isoform miniprotein binders to produce a tool for regenerative dentistry therapeutics and disease modeling goals.”

The corrected sentence appears below:

“Further, we apply the sci-RNA-seq predicted signaling pathways to generate a hiPSC derived odontoblast differentiation method (iOB) using *de novo* designed FGFR1/2 c-isoform mini protein binders to produce a tool for regenerative dentistry therapeutics and disease modeling goals.”

A correction has been made to **Materials and methods**, *2.2.4 De novo designed FGFR1/2c isoform mini binder expression*, Paragraph 1. This section previously stated:

“The sequences encoding the *de novo* designed fibroblast growth factor receptor-c (FGFR1/2c) isoform mini binder alone (hereby referred to as mb7) or fused to a hexameric scaffold (hereby referred to as C6) were synthesized and cloned into modified pET-29b(+) *E. coli* plasmid expression vectors (GenScript, N-terminal 8-His tag followed by a TEV cleavage site). The sequence of the N-terminal tag is MSHHHHHHHHSENLYFQSGGG, which is followed immediately by the sequence of the designed protein (31, 32). Plasmids were transformed into chemically competent *E. coli* Lemo21 cells (NEB). The protein expression was performed using Studier autoinduction medium supplemented with antibiotic, and cultures were grown overnight. Then, IPTG was added to a final concentration of 500 mM and the cells were grown overnight at 22°C for expression. The cells were collected by spinning at 4,000 *g* for 10 min and then resuspended in lysis buffer [300 mM NaCl, 30 mM Tris-HCL (pH 8.0), with 0.25% CHAPS for cell assay samples] with DNase and protease inhibitor tablets. The cells were lysed with a sonicator (Qsonica Sonicators) for 4 min in total (2 min each time, 10 s on, 10 s off) with an amplitude of 80%. The soluble fraction was clarified by centrifugation at 20,000 *g* for 30 min. The soluble fraction was purified by immobilized metal affinity chromatography (Qiagen) followed by FPLC SEC (Superdex 75 10/300 GL, GE Healthcare). The protein samples were characterized by SDS–PAGE, and purity was greater than 95%. Protein concentrations were determined by absorbance at 280 nm measured with a NanoDrop spectrophotometer (Thermo Fisher Scientific) using predicted extinction coefficients.”

The corrected section appears below:

“De novo designed fibroblast growth factor receptor-c (FGFR1/2c) isoform specific mini binder alone (hereby referred to as mb7) or fused to a hexameric scaffold (hereby referred to as C6) were produced as described previously (30,31,32).”

A correction has been made to **Materials and methods**, *2.2.5 iNC derived odontoblast differentiation*, Paragraph 1. This section previously stated:

“In order to fully elucidate the role of FGF signaling in odontoblast development we utilized the *de novo* designed FGFR1/2c isoform minibinders mb7,which functions as a FGF antagonist, and C6, which acts as a FGF agonist (30,31).”

The corrected section appears below:

“In order to fully elucidate the role of FGF signaling in odontoblast development we utilized mb7, which functions as a FGFR antagonist, and C6, which acts as a FGFR agonist (30–32).”

A correction has been made to **Results**, Paragraph 1. This sentence previously stated:

“Third, we utilize the information on the critical signaling pathways involved in human odontoblast development to produce a hiPSC derived odontoblast differentiation protocol (iOB) and dissect the role of FGF signaling using *de novo* designed FGFR1/2c isoform minibinders (30,31).”

The corrected sentence appears below:

“Third, we utilize the information on the critical signaling pathways involved in human odontoblast development to produce a hiPSC derived odontoblast differentiation protocol (iOB) and dissect the role of FGF signaling using *de novo* designed c-isoform specific FGFR1/2 minibinders (30–32).”

A correction has been made to **Results**, *3.5. Early FGF and BMP activation with late FGF agonism using the de novo designed FGFR1/2c isoform minibinder C6 and HH activation leads to more mature hiPSC derived odontoblast differentiation in vitro (iOB),* Paragraph 2. This sentence previously stated:

“In order to elucidate the role of FGF signaling in odontoblast differentiation, as predicted by sci-RNA-seq analysis, odontogenic medium was additionally supplemented with the *de novo* designed FGFR1/2c minibinder agonist C6 (iOBC6) (30, 31); C6 followed by the denovo design FGFR1/2c minibinder antagonist mb7 (iOB C6 to mb7) (30, 31) (Figure 5A); or basic FGF (iOB bFGF) (Figures 5B,C).”

The corrected sentence appears below:

“In order to elucidate the role of FGF signaling in odontoblast differentiation, as predicted by sci-RNa-seq analysis, odontogenic medium was additionally supplemented with C6 (iOb C6); C6 followed by mb7 (iOB C6 to mb7) (30–32) (Figure 5A); or basic FGF (iOB bFGF) (Figures 5B,C).”

A correction has been made to **Discussion**, *4.5. Single Cell RNA Sequencing Guided Targeting of FGFR1 C-Isoform Using De Novo Mini Binders Produces More Mature hiPSC Derived Odontoblasts In Vitro (iOB),* Paragraph 1. This paragraph previously stated:

“…Agonism of FGFR1/2c isoform using the *de novo* designed mini binder C6 produced the most advanced odontoblasts with significantly increased expression of mature odontoblast markers DSPP and DMP1 at both the RNA and protein levels, with significantly enhanced mineralization capacity (iOB C6)…These findings indicate that while sci-RNA-seq identified BMP and HH signaling play critical roles in early human odontoblast development, it is the agonism of FGF signaling using the *de novo* designed FGFR1/2c mini binder C6 that produces odontoblast with significantly greater maturation and biomineralization capacity, loss of which results in inhibited mineral deposition activity. “

The corrected sentence appears below:

“…Agonism of c-isoform FGFR1/2 using C6 produced the most advanced odontoblasts with significantly increased expression of mature odontoblast markers DSPP and DMP1 at both the RNA and protein levels, with significantly enhanced mineralization capacity (iOB C6). These findings indicate that while sci-RNA-seq identified BMP and HH signaling play critical roles in early human odontoblast development, it is the agonist of FGF signaling using the *de novo* designed c-isoform specific FGFR1/2 hexameric minibinder C6 that produces odontoblasts with significantly greater maturation and biomineralization capacity, loss of which results in inhibited mineral deposition activity.”

A correction has been made to **Conclusion**, Paragraph 1. This paragraph previously stated:

“This study marks the first application of artificial intelligence optimized proteins in the field of regenerative dentistry and provides a profound tool to be used for therapeutic and disease modeling goals. “

The corrected sentence appears below:

“This study marks the first application of *de novo* designed proteins in the field of regenerative dentistry and provides a profound tool to be used for therapeutic and disease modeling goals.”

The authors apologize for these errors and state that this does not change the scientific conclusions of the article in any way. The original article has been updated.

