# Supplementary figures and images for: Single cell RNA sequencing reveals human tooth type identity and guides in vitro hiPSC derived odontoblast differentiation (iOB)
Source: Front Dent Med. 2023 Jul 20;4:1209503. doi: 10.3389/fdmed.2023.1209503 (PMC10802932; doi:10.3389/fdmed.2023.1209503)

[illegible]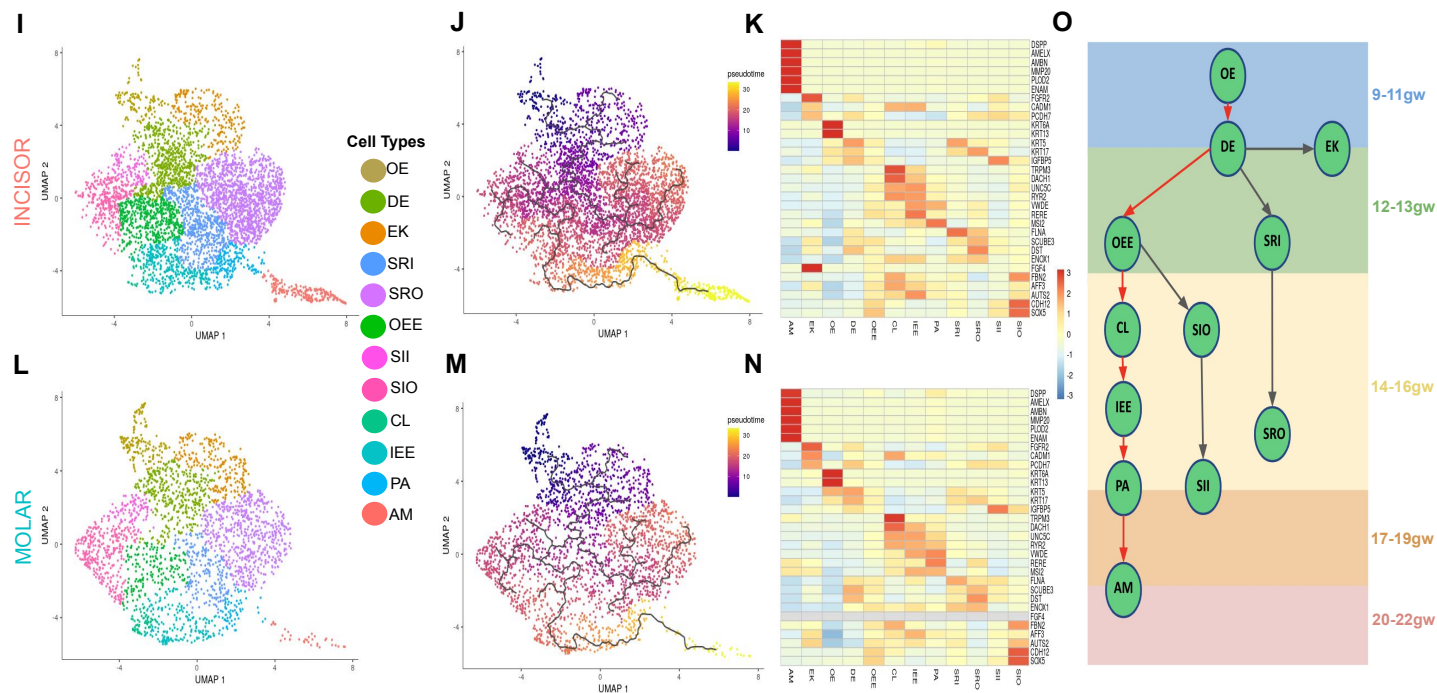

Supplement: Supplementary Figure S1 — A Single Cell Atlas of the Developing Human Incisor and Molar Dental Cell Types. (A) Downstream signaling pathways ranked by activity with detailed signaling ligands per pathway in odontoblast development indicate FGF and BMP are critical to the dental papilla (DP) as it transitions to preodontoblast (POB); HH, BMP, and NOTCH are the most active as POB transitions to odontoblast (OB). UMAP graph of subclustered incisor (B) and molar (E) tooth germ type dental mesenchyme derived cells from the total dataset identified conserved 6 transcriptionally unique clusters identified by collating highly expressed cluster-specific genes including dental papilla (DP), preodontoblast (POB), odontoblast (OB), subodontoblast (SOB), odontoblast (OB), dental ectomesenchyme (DEM), and dental follicle (DF). Pseudotime trajectory analysis for dental mesenchyme derived cells suggest two progenitors DP and DEM (blue), that give rise to differentiated OB (yellow) for both incisor (C) and molar (F). Heatmaps for putative marker genes for each dental mesenchyme cell type were produced for incisor (D) and molar (G). Simplified differentiation trajectory tree illustrating a common DEM progenitor gives rise to both DP and DF. In the OB lineage (red), DP gives rise to POB, followed by OB; DF lineage (grey) indicates DEM giving rise to DF, which gives rise to SOB, with a suggested transition through POB-like state before giving rise to OB (H). UMAP graph of subclustered incisor (I) and molar (L) tooth germ type dental epithelium derived cells from the total dataset yielded 12 unique clusters that we identified by collating highly expressed cluster-specific genes including: oral epithelium (OE), dental epithelium (DE), enamel knot (EK), inner and outer enamel epithelium (IEE, OEE), cervical loop (CL), inner and outer stratum intermedium (SII, SIO), inner and outer stellate reticulum (SRI, SRO), pre-ameloblasts (PA) and ameloblast (AM). Pseudotime trajectory analysis for dental epithelium derived c [file Image1.pdf]

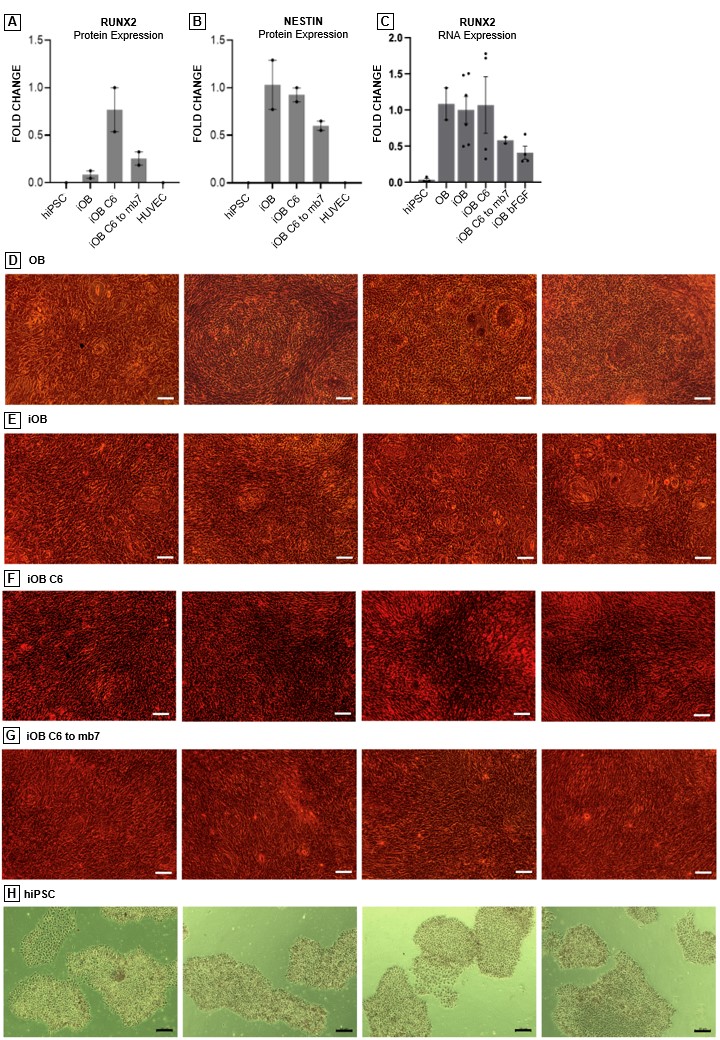

Supplement: Supplementary Figure S2 — C-Isoform Specific Activation of FGFR1 with De Novo Designed Mini Binder C6 Promotes Improved Mineralization Capacity of iOB. Quantification of Western Blot protein level of RUNX2 (A) and NESTIN (B). qPCR analysis of odontoblast marker RUNX2 (C). Extracellular calcifications assessed via Alizarin Red Stain (ARS) in iNC cultured in odontogenic medium (OB) (D); supplemented with BMP4 and SAG (iOB) (E); C6 (iOB C6) (F); C6 followed by mb7 (iOB C6 to mb7) (G); or undifferentiated hiPSC (H). Scale bar 20 µm. [file Image2.jpeg]
