# Supplementary material for: Single cell RNA sequencing reveals human tooth type identity and guides in vitro hiPSC derived odontoblast differentiation (iOB)
Source: Front Dent Med. 2023 Jul 20;4:1209503. doi: 10.3389/fdmed.2023.1209503 (PMC10802932; doi:10.3389/fdmed.2023.1209503)
Supplement: Supplementary file 3 [file Table1.pdf]

Supplemental Table 1. Sci-RNA-Seq Based Signaling Pathways Predicted to Guide Human Dental Papilla to Preodontoblast Transition.

Pathway	Pathway Activity	Percentage of Signaling Pathway Contribution to Overall Signaling Activity
FGF	0.553875393409193	22.5%
BMP	0.550038163958196	22.3%
NOTCH	0.196219363745902	8.0%
ROBO	0.183949373820091	7.5%
TGFb	0.140973592959099	5.7%
HH	0.139080487461313	5.6%
ACTIVIN	0.138046901967685	5.6%
BMP10	0.0945780656332932	3.8%
WNT	0.0918974676295192	3.7%
GDF	0.0862911705726348	3.5%
ncWNT	0.0734167231137553	3.0%
HGF	0.0504931236916422	2.0%
EGF	0.0304440629052946	1.2%
NODAL	0.0259444591030694	1.1%
IGF	0.0244637761693692	1.0%
NRG	0.0243552503242357	1.0%
VEGF	0.0158145446872169	0.6%
NT	0.013599439781608	0.6%
GDNF	0.0110040049089926	0.4%
PDGF	0.0105747846085393	0.4%
NGF	0.00679971989080399	0.3%
EDA	0.00208039892802572	0.1%
