# Supplementary material for: Single cell RNA sequencing reveals human tooth type identity and guides in vitro hiPSC derived odontoblast differentiation (iOB)
Source: Front Dent Med. 2023 Jul 20;4:1209503. doi: 10.3389/fdmed.2023.1209503 (PMC10802932; doi:10.3389/fdmed.2023.1209503)
Supplement: Supplementary file 4 [file Table2.pdf]

Supplemental Table 2. Sci-RNA-Seq Base[illegible] Dental Papilla to Pre[illegible]		
**Ligand**	**Pathway**	**Ligand Activity Rank**
GDF5	BMP	0.107936854104948
FGF2	FGF	0.0951687697643382
BMP10	BMP10	0.0945780656332932
SLIT2	ROBO	0.0919746869100457
INHBA	ACTIVIN	0.0861579837615462
TGFB3	TGFb	0.0824392399327761
WNT5A	ncWNT	0.0734167231137553
BMP15	BMP	0.0706731787268401
BMP2	BMP	0.0706731787268401
BMP4	BMP	0.0706731787268401
BMP6	BMP	0.0706731787268401
BMP7	BMP	0.0706731787268401
SLIT1	ROBO	0.0637033830817625
GDF9	GDF	0.0603467114695654
TGFB2	TGFb	0.0585343530263231
HGF	HGF	0.0504931236916422
GDF6	BMP	0.0481681205331745
DHH	HH	0.0463601624871043
IHH	HH	0.0463601624871043
SHH	HH	0.0463601624871043
FGF1	FGF	0.044675646072696
FGF4	FGF	0.0419522374212554
FGF7	FGF	0.0419522374212554
GDF7	BMP	0.0405672956858721
DLK1	NOTCH	0.0392438727491804
DLL1	NOTCH	0.0392438727491804
DLL4	NOTCH	0.0392438727491804
JAG1	NOTCH	0.0392438727491804
JAG2	NOTCH	0.0392438727491804
FGF10	FGF	0.0366807225255164
FGF18	FGF	0.0366807225255164
FGF19	FGF	0.0366807225255164
FGF23	FGF	0.0366807225255164
FGF3	FGF	0.0366807225255164
FGF5	FGF	0.0366807225255164
FGF6	FGF	0.0366807225255164
FGF8	FGF	0.0366807225255164
FGF9	FGF	0.0366807225255164
WNT3A	WNT	0.0345141279478644
SLIT3	ROBO	0.0282713038282832
GDF11	GDF	0.0259444591030694
INHBB	ACTIVIN	0.0259444591030694
INHBC	ACTIVIN	0.0259444591030694
NODAL	NODAL	0.0259444591030694
IGF2	IGF	0.0244637761693692

WNT1	WNT	0.0212302919460077
WNT7B	WNT	0.0212302919460077
WNT4	WNT	0.0149227557896393
PDGFB	PDGF	0.0105747846085393
NGF	NGF	0.00679971989080399
NTF3	NT	0.00679971989080399
NTF4	NT	0.00679971989080399
BTC	EGF	0.00608881258105893
EGF	EGF	0.00608881258105893
EREG	EGF	0.00608881258105893
HBEGF	EGF	0.00608881258105893
NRG1	NRG	0.00608881258105893
NRG2	NRG	0.00608881258105893
NRG3	NRG	0.00608881258105893
NRG4	NRG	0.00608881258105893
TGFA	EGF	0.00608881258105893
GDNF	GDNF	0.00550200245449629
NRTN	GDNF	0.00550200245449629
PGF	VEGF	0.00527151489573897
VEGFA	VEGF	0.00527151489573897
VEGFB	VEGF	0.00527151489573897
EDA	EDA	0.00208039892802572

d Signaling Ligands Predicted to Guide Human
eodontoblast Transition.

Percentage Contribution to Pathway Activity
4.4%
3.9%
3.8%
3.7%
3.5%
3.3%
3.0%
2.9%
2.9%
2.9%
2.9%
2.9%
2.6%
2.4%
2.4%
2.0%
2.0%
1.9%
1.9%
1.9%
1.8%
1.7%
1.7%
1.6%
1.6%
1.6%
1.6%
1.6%
1.6%
1.5%
1.5%
1.5%
1.5%
1.5%
1.5%
1.5%
1.5%
1.5%
1.4%
1.1%
1.1%
1.1%
1.1%
1.1%
1.0%

0.9%
0.9%
0.6%
0.4%
0.3%
0.3%
0.3%
0.2%
0.2%
0.2%
0.2%
0.2%
0.2%
0.2%
0.2%
0.2%
0.2%
0.2%
0.2%
0.2%
0.2%
0.1%
