# Supplementary material for: Single cell RNA sequencing reveals human tooth type identity and guides in vitro hiPSC derived odontoblast differentiation (iOB)
Source: Front Dent Med. 2023 Jul 20;4:1209503. doi: 10.3389/fdmed.2023.1209503 (PMC10802932; doi:10.3389/fdmed.2023.1209503)
Supplement: Supplementary file 5 [file Table3.pdf]

Supplemental Table 3. Sci-RNA-S

1	**Pathway**	**Pathway Activity**
2	HH	6.58091396461215
3	NOTCH	5.95770562015242
4	BMP	3.60889869037237
5	ROBO	2.61501748823247
6	GDF	1.97619555193484
7	WNT	1.89405110769015
8	ncWNT	1.34370262741258
9	ACTIVIN	1.21532494524332
10	FGF	1.05167605818864
11	NT	0.600320685338475
12	BMP10	0.545134512038139
13	HGF	0.430411617253077
14	NODAL	0.405108315081107
15	VEGF	0.309959146896494
16	NGF	0.171735189235851
17	TGFb	0.140026196957032
	EDA	0.00857715901768102

Seq Based Signaling Pathways Predicted to Guide Human Preodontoblast to Odontoblast Transition.
**Percentage of Signaling Pathway Contribution to Overall Signaling Activity**
22.8%
20.6%
12.5%
9.1%
6.8%
6.6%
4.7%
4.2%
3.6%
2.1%
1.9%
1.5%
1.4%
1.1%
0.6%
0.5%
0.0%
