# Supplementary material for: Single cell RNA sequencing reveals human tooth type identity and guides in vitro hiPSC derived odontoblast differentiation (iOB)
Source: Front Dent Med. 2023 Jul 20;4:1209503. doi: 10.3389/fdmed.2023.1209503 (PMC10802932; doi:10.3389/fdmed.2023.1209503)
Supplement: Supplementary file 6 [file Table4.pdf]

Supplemental Table 4. Sci-RNA-Seq Base[illegible] Preodontoblast to [illegible]		
**Ligand**	**Pathway**	**Ligand Activity**
DHH	HH	2.19363798820405
IHH	HH	2.19363798820405
SHH	HH	2.19363798820405
GDF9	GDF	1.57108723685373
WNT5A	ncWNT	1.34370262741258
SLIT2	ROBO	1.30750874411624
DLK1	NOTCH	1.19154112403048
DLL1	NOTCH	1.19154112403048
DLL4	NOTCH	1.19154112403048
JAG1	NOTCH	1.19154112403048
JAG2	NOTCH	1.19154112403048
SLIT3	ROBO	0.929926855276543
GDF5	BMP	0.908474659235602
WNT3A	WNT	0.840336283258083
FGF2	FGF	0.795237112993616
WNT4	WNT	0.734608917944184
BMP10	BMP10	0.545134512038139
HGF	HGF	0.430411617253077
BMP15	BMP	0.405108315081107
BMP2	BMP	0.405108315081107
BMP4	BMP	0.405108315081107
BMP5	BMP	0.405108315081107
BMP6	BMP	0.405108315081107
BMP7	BMP	0.405108315081107
GDF11	GDF	0.405108315081107
INHBA	ACTIVIN	0.405108315081107
INHBB	ACTIVIN	0.405108315081107
INHBC	ACTIVIN	0.405108315081107
NODAL	NODAL	0.405108315081107
SLIT1	ROBO	0.377581888839694
GDF7	BMP	0.269774140650125
BDNF	NT	0.200106895112825
NTF3	NT	0.200106895112825
NTF4	NT	0.200106895112825
NGF	NGF	0.171735189235851
WNT1	WNT	0.156057160151849
FGF1	FGF	0.153119229562863
TGFB3	TGFb	0.140026196957032
WNT3	WNT	0.105727365313899
FGF7	FGF	0.103319715632165
PGF	VEGF	0.103319715632165
VEGFA	VEGF	0.103319715632165
VEGFB	VEGF	0.103319715632165
WNT2	WNT	0.028660690511067
WNT7A	WNT	0.028660690511067

EDA	EDA	0.00857715901768102

d Signaling Ligands Predicted to Guide Human
Odontoblast Transition.

Percentage Contribution to Pathway Activity
7.6%
7.6%
7.6%
5.4%
4.7%
4.5%
4.1%
4.1%
4.1%
4.1%
4.1%
3.2%
3.1%
2.9%
2.8%
2.5%
1.9%
1.5%
1.4%
1.4%
1.4%
1.4%
1.4%
1.4%
1.4%
1.4%
1.4%
1.4%
1.4%
1.3%
0.9%
0.7%
0.7%
0.7%
0.6%
0.5%
0.5%
0.5%
0.4%
0.4%
0.4%
0.4%
0.4%
0.1%
0.1%

0.0%
