# Supplementary material for: Single cell RNA sequencing reveals human tooth type identity and guides in vitro hiPSC derived odontoblast differentiation (iOB)
Source: Front Dent Med. 2023 Jul 20;4:1209503. doi: 10.3389/fdmed.2023.1209503 (PMC10802932; doi:10.3389/fdmed.2023.1209503)
Supplement: Supplementary file 7 [file Table5.pdf]

Supplemental Table 5. Sci-RNA-Seq Based Signaling Pathways Predicted to Gu
Incisor and Molar Tooth Types.

Tooth Type	Pathway	Pathway Activity	Tooth Type Percentage Contribution to Specific Pathway Activity
Incisor	FGF	2,255913037	87.6%
	EGF	1,872398921	58.4%
	WNT	0,846116729	93.6%
	ncWNT	1,326447731	51.5%
	VEGF	1,830368464	35.2%
	BMP	2,035953161	25.4%
	NRG	0,917997253	50.8%
	NOTCH	0,434647859	95.7%
	ROBO	1,053725035	31.4%
	PDGF	0,294881323	86.0%
	HH	0,689141692	35.9%
	IGF	0,244168221	100.0%
	TGFb	0,384333209	38.7%
	HGF	0,20761884	69.5%
	ACTIVIN	0,232318742	49.3%
	NT	0,453300052	25.2%
	NGF	0,104938555	88.0%
	GDNF	0,110043294	53.6%
	GDF	0,037897673	100.0%
Molar	BMP	2,035953161	74.6%
	VEGF	1,830368464	64.8%
	EGF	1,872398921	41.6%
	ROBO	1,053725035	68.6%
	ncWNT	1,326447731	48.5%
	NRG	0,917997253	49.2%
	HH	0,689141692	64.1%
	NT	0,453300052	74.8%
	FGF	2,255913037	12.4%
	TGFb	0,384333209	61.3%
	ACTIVIN	0,232318742	50.7%
	HGF	0,20761884	30.5%
	WNT	0,846116729	6.4%
	GDNF	0,110043294	46.4%
	PDGF	0,294881323	14.0%
	NOTCH	0,434647859	4.3%
	NGF	0,104938555	12.0%

ide Human Enamel Knot Development In

Percentage of Signaling Pathway Contribution to Overall Signaling Activity
14,70%
12,20%
5,50%
8,70%
11,90%
13,30%
6,00%
2,80%
6,90%
1,90%
4,50%
1,60%
2,50%
1,40%
1,50%
3,00%
0,70%
0,70%
0,20%
13,30%
11,90%
12,20%
6,90%
8,70%
6,00%
4,50%
3,00%
14,70%
2,50%
1,50%
1,40%
5,50%
0,70%
1,90%
2,80%
0,70%
