# Supplementary material for: Single cell RNA sequencing reveals human tooth type identity and guides in vitro hiPSC derived odontoblast differentiation (iOB)
Source: Front Dent Med. 2023 Jul 20;4:1209503. doi: 10.3389/fdmed.2023.1209503 (PMC10802932; doi:10.3389/fdmed.2023.1209503)
Supplement: Supplementary file 8 [file Table6.pdf]

Supplemental Table 6. Sci-RNA-Seq Based Signaling
Enamel Knot Development In Incisor a

Tooth Type	Ligand	Pathway	Ligand Activity Rank
Incisor	WNT5A	ncWNT	0,683774217
	FGF2	FGF	0,500956631
	HBEGF	EGF	0,35206822
	FGF1	FGF	0,345910039
	WNT3A	WNT	0,322106244
	VEGFA	VEGF	0,307264919
	WNT3	WNT	0,280611821
	IGF2	IGF	0,196835297
	NRG1	NRG	0,187359507
	GDF5	BMP	0,18601366
	SHH	HH	0,18428887
	BTC	EGF	0,165658229
	EGF	EGF	0,165658229
	EREG	EGF	0,165658229
	NRG4	NRG	0,165658229
	TGFA	EGF	0,165658229
	SLIT1	ROBO	0,165416055
	SLIT2	ROBO	0,165416055
	WNT7B	WNT	0,154647727
	HGF	HGF	0,144213146
	FGF6	FGF	0,144130464
	PDGFC	PDGF	0,137250801
	VEGFB	VEGF	0,12240232
	FGF4	FGF	0,114502356
	FGF7	FGF	0,114502356
	PGF	VEGF	0,110020688
	VEGFC	VEGF	0,104307248
	FGF10	FGF	0,094443207
	FGF17	FGF	0,094443207
	FGF18	FGF	0,094443207
	FGF19	FGF	0,094443207
	FGF23	FGF	0,094443207
	FGF3	FGF	0,094443207
	FGF5	FGF	0,094443207
	FGF9	FGF	0,094443207
	NGF	NGF	0,092313747
	NRG2	NRG	0,087374536
	DLK1	NOTCH	0,083160729
	DLL1	NOTCH	0,083160729
	DLL4	NOTCH	0,083160729
	JAG1	NOTCH	0,083160729
	JAG2	NOTCH	0,083160729
	TGFB2	TGFb	0,081282736

AREG	EGF	0,078283693
BMP2	BMP	0,070723474
BMP7	BMP	0,070723474
TGFB3	TGFb	0,067599808
INHBA	ACTIVIN	0,063012809
DHH	HH	0,062919301
BMP4	BMP	0,06054965
BMP5	BMP	0,06054965
BMP6	BMP	0,06054965
INHBC	ACTIVIN	0,051580601
IGF1	IGF	0,047332924
PDGFB	PDGF	0,045880031
NTF3	NT	0,039723125
NTF4	NT	0,039723125
GDF11	GDF	0,037897673
PDGFA	PDGF	0,035303021
PDGFD	PDGF	0,035303021
BDNF	NT	0,034859891
WNT2	WNT	0,034738824
NRG3	NRG	0,026039389
ARTN	GDNF	0,0147411
GDNF	GDNF	0,0147411
NRTN	GDNF	0,0147411
PSPN	GDNF	0,0147411
GDF6	BMP	0,008969048
WNT5A1	ncWNT	0,642673514
GDF51	BMP	0,460417533
PGF1	VEGF	0,381742947
VEGFA1	VEGF	0,381742947
SLIT21	ROBO	0,361446463
VEGFC1	VEGF	0,317773127
DHH1	HH	0,220966761
SHH1	HH	0,220966761
SLIT11	ROBO	0,218053587
HBEGF1	EGF	0,177326636
FGF21	FGF	0,152425898
BMP21	BMP	0,151065289
BMP41	BMP	0,151065289
BMP51	BMP	0,151065289
BMP61	BMP	0,151065289
BMP71	BMP	0,151065289
GDF61	BMP	0,151065289
GDF7	BMP	0,151065289
BTC1	EGF	0,150521864
EGF1	EGF	0,150521864
EREG1	EGF	0,150521864
NRG11	NRG	0,150521864
NRG21	NRG	0,150521864

Molar	NRG41	NRG	0,150521864
	TGFA1	EGF	0,150521864
	SLIT3	ROBO	0,143392876
	BDNF1	NT	0,121414509
	INHBA1	ACTIVIN	0,117725332
	TGFB21	TGFb	0,117725332
	TGFB31	TGFb	0,117725332
	NTF31	NT	0,108789701
	NTF41	NT	0,108789701
	VEGFB1	VEGF	0,105114268
	FGF11	FGF	0,06396982
	FGF71	FGF	0,06396982
	HGF1	HGF	0,063405694
	PDGFC1	PDGF	0,041144447
	WNT7A	WNT	0,027006056
	WNT7B1	WNT	0,027006056
	ARTN1	GDNF	0,012769723
	GDNF1	GDNF	0,012769723
	NRTN1	GDNF	0,012769723
	PSPN1	GDNF	0,012769723
	NGF1	NGF	0,012624808
	DLK11	NOTCH	0,003768843
	DLL11	NOTCH	0,003768843
	DLL41	NOTCH	0,003768843
	JAG11	NOTCH	0,003768843
	JAG21	NOTCH	0,003768843
	WNT3A1	WNT	0

Ligands Predicted to Guide Human nd Molar Tooth Types.
**Percentage Contribution of Specific Ligand to Pathway Activity**
4,50%
3,30%
2,30%
2,30%
2,10%
2,00%
1,80%
1,30%
1,20%
1,20%
1,20%
1,10%
1,10%
1,10%
1,10%
1,10%
1,10%
1,10%
1,00%
0,90%
0,90%
0,90%
0,80%
0,70%
0,70%
0,70%
0,70%
0,60%
0,60%
0,60%
0,60%
0,60%
0,60%
0,60%
0,60%
0,60%
0,60%
0,50%
0,50%
0,50%
0,50%
0,50%
0,50%

0,50%
0,50%
0,50%
0,40%
0,40%
0,40%
0,40%
0,40%
0,40%
0,30%
0,30%
0,30%
0,30%
0,30%
0,20%
0,20%
0,20%
0,20%
0,20%
0,20%
0,10%
0,10%
0,10%
0,10%
0,10%
4,20%
3,00%
2,50%
2,50%
2,40%
2,10%
1,40%
1,40%
1,40%
1,20%
1,00%
1,00%
1,00%
1,00%
1,00%
1,00%
1,00%
1,00%
1,00%
1,00%
1,00%
1,00%
1,00%

1,00%
1,00%
0,90%
0,80%
0,80%
0,80%
0,80%
0,70%
0,70%
0,70%
0,40%
0,40%
0,40%
0,30%
0,20%
0,20%
0,10%
0,10%
0,10%
0,10%
0,10%
0,00%
0,00%
0,00%
0,00%
0,00%
0,00%
