# Supplementary material for: Single cell RNA sequencing reveals human tooth type identity and guides in vitro hiPSC derived odontoblast differentiation (iOB)
Source: Front Dent Med. 2023 Jul 20;4:1209503. doi: 10.3389/fdmed.2023.1209503 (PMC10802932; doi:10.3389/fdmed.2023.1209503)
Supplement: Supplementary file 9 [file Table7.pdf]

Supplemental Table 7. Sci-RNA-Seq Based Signaling Pathways Predicted to G
Enamel Epithelium In Incisor and Molar Tooth

Tooth Type	Pathway	Pathway Activity	Tooth Type Percentage Contribution to Specific Pathway Activity
Incisor	FGF	1,959470652	66.0%
	EGF	1,281646299	65.1%
	NRG	0,488999826	63.4%
	BMP	0,265097665	100.0%
	WNT	0,059208122	100.0%
	IGF	0,058180267	100.0%
	NT	0,044676839	100.0%
	TGFb	0,096107525	40.0%
	VEGF	0,034280165	100.0%
	ncWNT	0,029604061	100.0%
	PDGF	0,028400044	100.0%
	HGF	0,027757206	100.0%
	NGF	0,022338419	100.0%
Molar	FGF	1,959470652	34.0%
	EGF	1,281646299	34.9%
	NRG	0,488999826	36.6%
	HH	0,111596452	100.0%
	TGFb	0,096107525	60.0%

…uide Human Dental Epithelium to Outer … Types.

Percentage of Signaling Pathway Contribution to Overall Signaling Activity
43,50%
28,40%
10,80%
5,90%
1,30%
1,30%
1,00%
2,10%
0,80%
0,70%
0,60%
0,60%
0,50%
43,50%
28,40%
10,80%
2,50%
2,10%
