# Supplementary material for: Single cell RNA sequencing reveals human tooth type identity and guides in vitro hiPSC derived odontoblast differentiation (iOB)
Source: Front Dent Med. 2023 Jul 20;4:1209503. doi: 10.3389/fdmed.2023.1209503 (PMC10802932; doi:10.3389/fdmed.2023.1209503)
Supplement: Supplementary file 10 [file Table8.pdf]

Supplemental Table 8. Sci-RNA-Seq Based Signaling Ligands Predicted to Guide Human Dental Epithelium to Outer Enamel Epithelium In Incisor and Molar Tooth Types.				
**Tooth Type**	**Ligand**	**Pathway**	**Ligand Activity Rank**	**Percentage Contribution of Specific Ligand to Pathway Activity**
Incisor	BTC	EGF	0,156372361	3,50%
	EGF	EGF	0,156372361	3,50%
	EREG	EGF	0,156372361	3,50%
	TGFA	EGF	0,156372361	3,50%
	FGF17	FGF	0,12927155	3,50%
	FGF18	FGF	0,12927155	3,50%
	FGF19	FGF	0,12927155	3,50%
	FGF2	FGF	0,12927155	3,50%
	FGF23	FGF	0,12927155	3,50%
	FGF3	FGF	0,12927155	3,50%
	FGF4	FGF	0,12927155	3,50%
	FGF5	FGF	0,12927155	3,50%
	FGF6	FGF	0,12927155	3,50%
	FGF9	FGF	0,12927155	3,50%
	AREG	EGF	0,106077218	3,50%
	NRG1	NRG	0,103548192	3,50%
	NRG2	NRG	0,103548192	3,50%
	HBEGF	EGF	0,103119312	3,50%
	NRG4	NRG	0,103119312	3,50%
	IGF2	IGF	0,058180267	3,50%
	TGFB3	TGFb	0,038403654	3,50%
	BMP2	BMP	0,037871095	3,50%
	BMP4	BMP	0,037871095	3,50%
	BMP5	BMP	0,037871095	3,50%
	BMP6	BMP	0,037871095	3,50%
	BMP7	BMP	0,037871095	3,50%
	GDF5	BMP	0,037871095	3,50%
	GDF6	BMP	0,037871095	3,50%
	WNT2	WNT	0,029604061	3,50%
	WNT3	WNT	0,029604061	3,50%
	WNT5A	ncWNT	0,029604061	3,50%
	HGF	HGF	0,027757206	3,50%
	NGF	NGF	0,022338419	3,50%
	NTF3	NT	0,022338419	3,50%
	NTF4	NT	0,022338419	3,50%
	VEGFB	VEGF	0,017140082	3,50%
	VEGFC	VEGF	0,017140082	3,50%
	PDGFA	PDGF	0,014200022	3,50%
	PDGFD	PDGF	0,014200022	3,50%
	BTC1	EGF	0,089392065	3,50%
	EGF1	EGF	0,089392065	3,50%
	EREG1	EGF	0,089392065	3,50%
	HBEGF1	EGF	0,089392065	3,50%

Molar	NRG21	NRG	0,089392065	3,50%
	NRG41	NRG	0,089392065	3,50%
	TGFA1	EGF	0,089392065	3,50%
	SHH	HH	0,078513275	3,50%
	FGF1	FGF	0,060614105	3,50%
	FGF10	FGF	0,060614105	3,50%
	FGF171	FGF	0,060614105	3,50%
	FGF181	FGF	0,060614105	3,50%
	FGF191	FGF	0,060614105	3,50%
	FGF21	FGF	0,060614105	3,50%
	FGF231	FGF	0,060614105	3,50%
	FGF31	FGF	0,060614105	3,50%
	FGF61	FGF	0,060614105	3,50%
	FGF7	FGF	0,060614105	3,50%
	FGF91	FGF	0,060614105	3,50%
	TGFB31	TGFb	0,057703871	3,50%
	DHH	HH	0,033083177	3,50%
