# Supplementary material for: Single cell RNA sequencing reveals human tooth type identity and guides in vitro hiPSC derived odontoblast differentiation (iOB)
Source: Front Dent Med. 2023 Jul 20;4:1209503. doi: 10.3389/fdmed.2023.1209503 (PMC10802932; doi:10.3389/fdmed.2023.1209503)
Supplement: Supplementary file 11 [file Table9.pdf]

Supplemental Table 9. Sci-RNA-Seq Based Signaling Pathways Predicted to Gu[illegible] Preameloblast In Incisor and Molar Tooth Ty[illegible]

Tooth Type	Pathway	Pathway Activity	Tooth Type Percentage Contribution to Specific Pathway Activity
Incisor	BMP	2,44171437	46.3%
	FGF	2,013337036	19.2%
	ACTIVIN	0,231458237	57.7%
	IGF	0,081332796	100.0%
	GDF	0,139658607	46.7%
	TGFb	0,08557881	71.3%
	WNT	0,200906287	30.3%
	EGF	0,324009056	17.5%
	PDGF	0,230347276	22.9%
	ncWNT	0,115150982	25.8%
	NRG	0,110546108	25.8%
	HGF	0,019065557	100.0%
	HH	0,14319908	13.3%
	NT	0,014844845	19.1%
	ROBO	0,001859479	100.0%
	NGF	0,007808611	6.8%
	GDNF	0,508250825	0.0%
	VEGF	0	NA
Molar	FGF	2,013337036	80.8%
	BMP	2,44171437	53.7%
	GDNF	0,508250825	100.0%
	EGF	0,324009056	82.5%
	PDGF	0,230347276	77.1%
	WNT	0,200906287	69.7%
	HH	0,14319908	86.7%
	ACTIVIN	0,231458237	42.3%
	EDA	0,096809681	100.0%
	ncWNT	0,115150982	74.2%
	NRG	0,110546108	74.2%
	GDF	0,139658607	53.3%
	TGFb	0,08557881	28.7%
	NT	0,014844845	80.9%
	NGF	0,007808611	93.2%

uide Human Outer Enamel Epithelium to ypes.

Percentage of Signaling Pathway Contribution to Overall Signaling Activity
36,10%
29,80%
3,40%
1,20%
2,10%
1,30%
3,00%
4,80%
3,40%
1,70%
1,60%
0,30%
2,10%
0,20%
0,00%
0,10%
7,50%
0,00%
29,80%
36,10%
7,50%
4,80%
3,40%
3,00%
2,10%
3,40%
1,40%
1,70%
1,60%
2,10%
1,30%
0,20%
0,10%
