# Supplementary material for: Single cell RNA sequencing reveals human tooth type identity and guides in vitro hiPSC derived odontoblast differentiation (iOB)
Source: Front Dent Med. 2023 Jul 20;4:1209503. doi: 10.3389/fdmed.2023.1209503 (PMC10802932; doi:10.3389/fdmed.2023.1209503)
Supplement: Supplementary file 12 [file Table10.pdf]

Supplemental Table 10. Sci-RNA-Seq Based Signaling Lig
Enamel Epithelium to Preameloblast In Incis

Tooth Type	Ligand	Pathway	Ligand Activity Rank
Incisor	BMP2	BMP	0,171190463
	BMP4	BMP	0,171190463
	BMP5	BMP	0,171190463
	BMP6	BMP	0,171190463
	BMP7	BMP	0,171190463
	GDF5	BMP	0,171190463
	INHBC	ACTIVIN	0,133583034
	GDF6	BMP	0,102264257
	IGF2	IGF	0,081332796
	GDF11	GDF	0,065257733
	TGFB3	TGFb	0,060994862
	WNT2	WNT	0,030481812
	WNT3	WNT	0,030481812
	WNT11	ncWNT	0,029719839
	FGF1	FGF	0,028534704
	FGF10	FGF	0,028534704
	FGF17	FGF	0,028534704
	FGF18	FGF	0,028534704
	FGF19	FGF	0,028534704
	FGF2	FGF	0,028534704
	FGF23	FGF	0,028534704
	FGF3	FGF	0,028534704
	FGF4	FGF	0,028534704
	FGF5	FGF	0,028534704
	FGF6	FGF	0,028534704
	FGF7	FGF	0,028534704
	FGF9	FGF	0,028534704
	HGF	HGF	0,019065557
	DHH	HH	0,019051105
	PDGFA	PDGF	0,017561369
	PDGFB	PDGF	0,017561369
	PDGFD	PDGF	0,017561369
	FGF22	FGF	0,015195106
	BTC	EGF	0,011347987
	EGF	EGF	0,011347987
	EREG	EGF	0,011347987
	NRG1	NRG	0,011347987
	NRG2	NRG	0,011347987
	TGFA	EGF	0,011347987
	HBEGF	EGF	0,005858514
	NRG4	NRG	0,005858514
	AREG	EGF	0,005489474
	NTF3	NT	0,001417699

	NTF4	NT	0,001417699
	SLIT1	ROBO	0,000935587
	SLIT3	ROBO	0,000923892
	NGF	NGF	0,000527938
	ARTN	GDNF	0
	GDNF	GDNF	0
	NRTN	GDNF	0
	PSPN	GDNF	0
	VEGFC	VEGF	0
	WNT7A	WNT	0
Molar	BMP21	BMP	0,205207401
	BMP41	BMP	0,205207401
	BMP51	BMP	0,205207401
	BMP61	BMP	0,205207401
	BMP71	BMP	0,205207401
	GDF51	BMP	0,205207401
	ARTN1	GDNF	0,127062706
	GDNF1	GDNF	0,127062706
	NRTN1	GDNF	0,127062706
	PSPN1	GDNF	0,127062706
	FGF11	FGF	0,125168522
	FGF101	FGF	0,125168522
	FGF171	FGF	0,125168522
	FGF181	FGF	0,125168522
	FGF191	FGF	0,125168522
	FGF21	FGF	0,125168522
	FGF231	FGF	0,125168522
	FGF31	FGF	0,125168522
	FGF41	FGF	0,125168522
	FGF51	FGF	0,125168522
	FGF61	FGF	0,125168522
	FGF71	FGF	0,125168522
	FGF91	FGF	0,125168522
	WNT7A1	WNT	0,109363835
	INHBC1	ACTIVIN	0,097875203
	EDA	EDA	0,096809681
	PDGFA1	PDGF	0,088831584
	PDGFD1	PDGF	0,088831584
	WNT5A	ncWNT	0,085431143
	GDF7	BMP	0,081062927
	GDF111	GDF	0,074400873
	DHH1	HH	0,062073988
	SHH	HH	0,062073988
	BTC1	EGF	0,053453824
	EGF1	EGF	0,053453824
	EREG1	EGF	0,053453824
	NRG21	NRG	0,053453824
	TGFA1	EGF	0,053453824

	HBEGF1	EGF	0,028537795
	NRG41	NRG	0,028537795
	AREG1	EGF	0,024916029
	TGFB31	TGFb	0,024583949
	NTF31	NT	0,012009448
	WNT21	WNT	0,010192942
	WNT31	WNT	0,010192942
	WNT3A	WNT	0,010192942
	NGF1	NGF	0,007280673

ands Predicted to Guide Human Outer
or and Molar Tooth Types.

Percentage Contribution of Specific Ligand to Pathway Activity
2,50%
2,50%
2,50%
2,50%
2,50%
2,50%
2,00%
1,50%
1,20%
1,00%
0,90%
0,50%
0,50%
0,40%
0,40%
0,40%
0,40%
0,40%
0,40%
0,40%
0,40%
0,40%
0,40%
0,40%
0,40%
0,40%
0,40%
0,30%
0,30%
0,30%
0,30%
0,30%
0,20%
0,20%
0,20%
0,20%
0,20%
0,20%
0,20%
0,10%
0,10%
0,10%
0,00%

0,00%
0,00%
0,00%
0,00%
0,00%
0,00%
0,00%
0,00%
0,00%
0,00%
3,00%
3,00%
3,00%
3,00%
3,00%
3,00%
1,90%
1,90%
1,90%
1,90%
1,80%
1,80%
1,80%
1,80%
1,80%
1,80%
1,80%
1,80%
1,80%
1,80%
1,80%
1,80%
1,80%
1,60%
1,40%
1,40%
1,30%
1,30%
1,30%
1,20%
1,10%
0,90%
0,90%
0,80%
0,80%
0,80%
0,80%
0,80%

0,40%
0,40%
0,40%
0,40%
0,20%
0,20%
0,20%
0,20%
0,10%
